# Affinity maturation of the RLIP76 Ral binding domain to inform the design of stapled peptides targeting the Ral GTPases

**DOI:** 10.1074/jbc.RA120.015735

**Published:** 2020-11-23

**Authors:** Catherine A. Hurd, Paul Brear, Jefferson Revell, Sarah Ross, Helen R. Mott, Darerca Owen

**Affiliations:** 1 Department of Biochemistry, University of Cambridge, Cambridge, UK; 2 AstraZeneca, Sir Aaron Klug Building, Granta Park, Cambridge, UK; 3 Research and Early Development, Oncology R&D, AstraZeneca, Cambridge, UK

**Keywords:** cancer, cell signaling, chemical biology, drug discovery, GTPase, K-Ras, protein-protein interaction, Ral, RLIP76, stapled peptides, CD, circular dichroism, CPP, cell-penetrating peptide, DMF, *N,N*-dimethylformamide, FAM, carboxyfluorescein, Fmoc, 9-fluorenylmethoxycarbonyl, FP, fluorescence polarization, GAP, GTPase activating protein, GEF, guanine nucleotide exchange factor, GGTase, geranylgeranyltransferase, GMPPNP, guanosine 5'-[β,γ-imido] triphosphate, ITC, isothermal titration calorimetry, PPI, protein–protein interaction, RBD, Ral binding domain, SPA, scintillation proximity assay, TFA, trifluoroacetic acid

## Abstract

Ral GTPases have been implicated as critical drivers of cell growth and metastasis in numerous Ras-driven cancers. We have previously reported stapled peptides, based on the Ral effector RLIP76, that can disrupt Ral signaling. Stapled peptides are short peptides that are locked into their bioactive form using a synthetic brace. Here, using an affinity maturation of the RLIP76 Ral-binding domain, we identified several sequence substitutions that together improve binding to Ral proteins by more than 20-fold. Hits from the selection were rigorously analyzed to determine the contributions of individual residues and two 1.5 Å cocrystal structures of the tightest-binding mutants in complex with RalB revealed key interactions. Insights gained from this maturation were used to design second-generation stapled peptides based on RLIP76 that exhibited vastly improved selectivity for Ral GTPases when compared with the first-generation lead peptide. The binding of second-generation peptides to Ral proteins was quantified and the binding site of the lead peptide on RalB was determined by NMR. Stapled peptides successfully competed with multiple Ral–effector interactions in cellular lysates. Our findings demonstrate how manipulation of a native binding partner can assist in the rational design of stapled peptide inhibitors targeting a protein–protein interaction.

Ras proteins (H-Ras, N-Ras, and K-Ras) are well known as the most commonly mutated oncoproteins in human cancer, with activating mutations found in approximately 20% of cancers and with higher incidences in pancreatic (88%), colorectal (55%) and lung cancers (33%) (1). These small GTPases exist in two distinct conformations: an inactive GDP-bound form and an active GTP-bound form in which they can bind and signal through downstream effector proteins. Through this mechanism they have the ability to act as molecular switches. Ras signaling is “switched on” by proteins known as Guanine Exchange Factors (GEFs) and “switched off” by GTPase Activating Proteins (GAPs, reviewed in (2)).

Most Ras mutations found in cancer render the protein constitutively active, in the GTP-bound state. This leads to deregulated signaling through downstream effector pathways, resulting in uncontrolled cell growth and proliferation. Owing to the prevalence of their activation in cancer, Ras proteins have been the subject of intense therapeutic targeting for the past 4 decades but, until recently, the lack of compounds to target Ras proteins directly meant that they were considered “undruggable” (for reviews see (3, 4)). Several features of Ras proteins made them difficult targets: for instance, Ras proteins interact with their downstream effectors *via* protein–protein interactions (PPIs), which utilize large shallow surfaces that are often not amenable to targeting with small molecules. The nucleotide binding site on Ras, which would be an obvious point for small-molecule intervention, has not been a viable target owing to the high intracellular concentrations of GTP and a picomolar binding affinity of the nucleotide. Early attempts to block the posttranslational processing of Ras, which is required for membrane association and subsequent activity, proved unsuccessful due to compensatory processing by an alternative mechanism (5, 6). There has, however, been recent success in the direct inhibition of the K-Ras G12C mutant commonly found in lung cancers: Shokat and colleagues identified a binding pocket proximal to cysteine 12 that could be exploited to develop covalent inhibitors targeting this reactive residue (7). Several pharmaceutical companies have since reported their own candidates utilizing this approach that are currently being tested in the clinic (8, 9).

Difficulties in targeting Ras directly have resulted in considerable attempts to target effector pathways downstream of Ras. Of these, the Raf, PI3Kinase and RalGDS-Ral pathways are the best characterized, and indeed many Raf and PI3K pathway inhibitors exist in the clinic (10, 11). These inhibitors can be used to treat certain cancers but feedback mechanisms leading to the activation of other effector pathways and a narrow therapeutic window have meant that their efficacy in Ras-mutant cancers is limited (12). Combination trials to assess the efficacy of blocking components from both pathways are currently under way (13, 14, 15, 16); however, preliminary results have suggested that these combinations are too toxic (17, 18).

The third effector pathway downstream of Ras, the RalGDS-Ral pathway, has not yet been successfully targeted therapeutically despite growing evidence implicating this pathway as a critical mediator of survival in several Ras-mutant cancers, including pancreatic, colorectal, bladder, and melanoma (19). Upon association with and activation by Ras·GTP, RalGDS activates the Ral (Ras-like) small GTPases, RalA, and RalB. These are 206 amino acid proteins with 82% overall sequence identity and 100% identity in their effector binding regions. The Ral GTPases share a panel of effectors including RLIP76 (also called RalBP1), members of the exocyst complex (Sec5 and Exo84), and the transcription factor ZONAB. Despite their high degree of similarity, the Ral proteins exhibit divergent and contrasting roles in signaling and therefore in cancer development (20). These differences are partly brought about by their flexible C-terminal hypervariable regions, which are the sites of lipidation and other posttranslational modifications and dictate the site of membrane interaction, resulting in differential subcellular localization of RalA and RalB (21).

Early evidence of the critical role of Ral proteins in Ras-driven cancer came from Counter *et al.*, who demonstrated that activation of the RalGDS-Ral signaling pathway alone, and not Raf or PI3Kinase, was potent in transforming human cells (22). Constitutively activated RalA has been shown to be required for anchorage-independent growth of cancer cells (23, 24, 25), while RalB plays a role in invasion, metastasis (26) and the avoidance of apoptosis in tumor cells (27, 28). However, proliferation of noncancerous cells is unaffected by RalB activity (24). Inhibition of Ral–effector interactions can alleviate these effects as overexpression of a minimal Ral-binding domain (RBD) from RLIP76, which competes for effector binding, reduced anchorage-independent growth (24).

The Ral proteins share the same structural fold as Ras proteins and are therefore expected to be equally challenging to target using small molecules. However, several early studies managed to identify small-molecule inhibitors of the Ral GTPases. Yan *et al.* (29) used *in silico* screening to identify small-molecule inhibitors targeting a previously unidentified shallow binding pocket in the GDP-bound form of Ral, thereby stabilizing the inactive form and preventing Ral activation. Covalent inhibitors of Ral proteins have also been investigated: Meroueh and colleagues recently reported an inhibitor that was able to modify tyrosine 82 on Ral and subsequently inhibited GEF-mediated activation (30).

Stapled peptides have emerged in recent years as promising therapeutic tools for targeting PPIs mediated by α-helices. The technique, developed by Verdine *et al.*, involves the introduction of unnatural olefin-bearing amino acids at *i*, *i* + 4/7 positions, which are covalently linked by Grubbs’ ring closing metathesis (31). These chemical staples can induce and stabilize a helical structure and also impart other beneficial properties including cell penetration, increased proteolytic stability and improved binding affinity (31, 32). This approach has been successfully applied to target several small GTPases (reviewed in (33)).

We have previously reported the use of stapled peptides to inhibit Ral–effector interactions (34). Our solution structure of the RBD of RLIP76 in complex with RalB revealed that the interaction is mediated by a well-structured coiled-coil domain in which more than 80% of the interactions with Ral are mediated by the C-terminal α2 helix (35). As several effectors share a common binding site on Ral (36), we postulated that a peptide based on the RLIP76 RBD would be able to inhibit multiple Ral–effector interactions. We produced a series of stapled peptides spanning the RLIP76 RBD in which several staple lengths and positions were assessed (34). This work led to the identification of a stapled peptide based on the α2 helix of the RLIP76 RBD, which was able to inhibit multiple Ral–effector interactions, enter cells and inhibit autophagy, a RalB-dependent process. The peptide was selective for active RalB over the closely related GTPase R-Ras.

Here, we have used the RLIP76 RBD as a tool to guide the design of second-generation stapled peptides targeting the Ral GTPases. A CIS display selection using the RLIP76 RBD was performed to identify modified sequences with improved affinity for Ral proteins. These sequence alterations improved the affinity of stapled peptides based on the RLIP76 RBD in a similar manner. Investigations using the stapled peptide identified in previous work revealed issues relating to poor solubility and nonspecific binding, which have been addressed here, resulting in the development of stapled peptides with vastly improved solubility and selectivity for the active form of Ral proteins over several closely related small GTPases.

## Results

### CIS display maturation of the RLIP76 RBD

**Figure 1:**
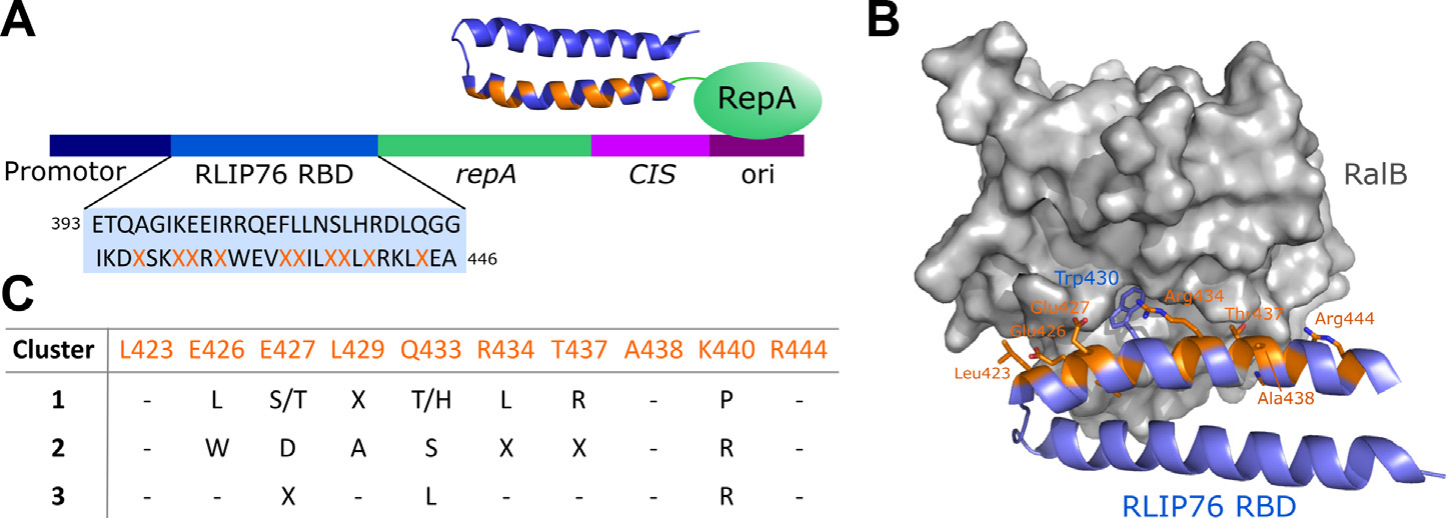
**CIS display maturation of the RLIP76 RBD.***A*, schematic of CIS display (37). The CIS display constructs comprised the RLIP76 RBD library fused to the gene encoding RepA. The positions that were allowed to alter during the selection are shown as *orange* Xs. *B*, structure of RalB in complex with the RLIP76 RBD (393–446, PDB ID: 2KWI). Residues of the RLIP76 RBD that were allowed to alter during the CIS display selections are shown as *orange sticks*: all of these residues were located within the α2 helix and were proximal to the Ral-binding interface. Trp430 is shown as *blue sticks* and was retained during all selections as it is known to be critical for binding to Ral proteins. *C*, sequence clusters identified with the highest frequencies in the selections. The residue positions in the RLIP76 RBD are listed. “-“ denotes no sequence change, and “X” denotes that a variety of amino acids were found at the position.

A series of CIS display selections (37) were carried out to identify potential sequence changes within our lead peptide sequence, which could improve affinity for Ral proteins (Fig. 1*A*). In CIS display, the variant library is fused to a gene encoding RepA, which captures the DNA from which it was translated upon recognition of a *cis* element, coupling the genetic material to the library. Up to 10^14^ sequences can be assessed by this method. As the unnatural amino acids utilized in the lead stapled peptide could not be included in an *in vitro* selection by conventional methods, the RLIP76 RBD (residues 393–446) was used as a model for peptide binding. During the selections, only residues that were contained in the lead peptide and were proximal to the Ral binding interface were permitted to alter (Fig. 1*B*). Trp430 was retained as this residue was known to be critical for Ral binding (36). Biotinylated RalA and RalB were immobilized in separate selections to allow for the identification of sequences that discriminated between the two proteins. After four rounds of biopanning, enrichment of binding sequences was relatively low compared with similar selections (38). This phenomenon may indicate that the parent sequence has already achieved close to an evolutionary maximum binding capacity using proteinogenic amino acids. The sequences obtained were largely similar between selections involving RalA and RalB, reflecting the high sequence identity of these proteins. Three consensus sequences were identified across multiple selections (Fig. 1*C*) and represented the most enriched sequences from the selections.

### Biophysical analysis of CIS display sequences

We reasoned that, while the consensus sequences represented an amalgamation of sequences, subtle residue cooperation within individual selected sequences might be required for high-affinity binding. Therefore, a selection of individual sequences that appeared with the highest frequency for each of the three major clusters were produced recombinantly in *E. coli* to quantify their binding to Ral proteins. Sequences containing the K440P mutation (Cluster 3, Fig. 1*C*) could not be produced due to protein precipitation during purification: it is likely that the helix-breaking Pro residue disrupts the structure of the protein leading to instability. The consensus sequence lacking the K440P substitution (E426L/E427T/Q433T/R434L/T437R) was produced as a His-tagged construct but did not bind to RalA in direct scintillation proximity assays (SPAs) (Fig. S1).

**Figure 2:**
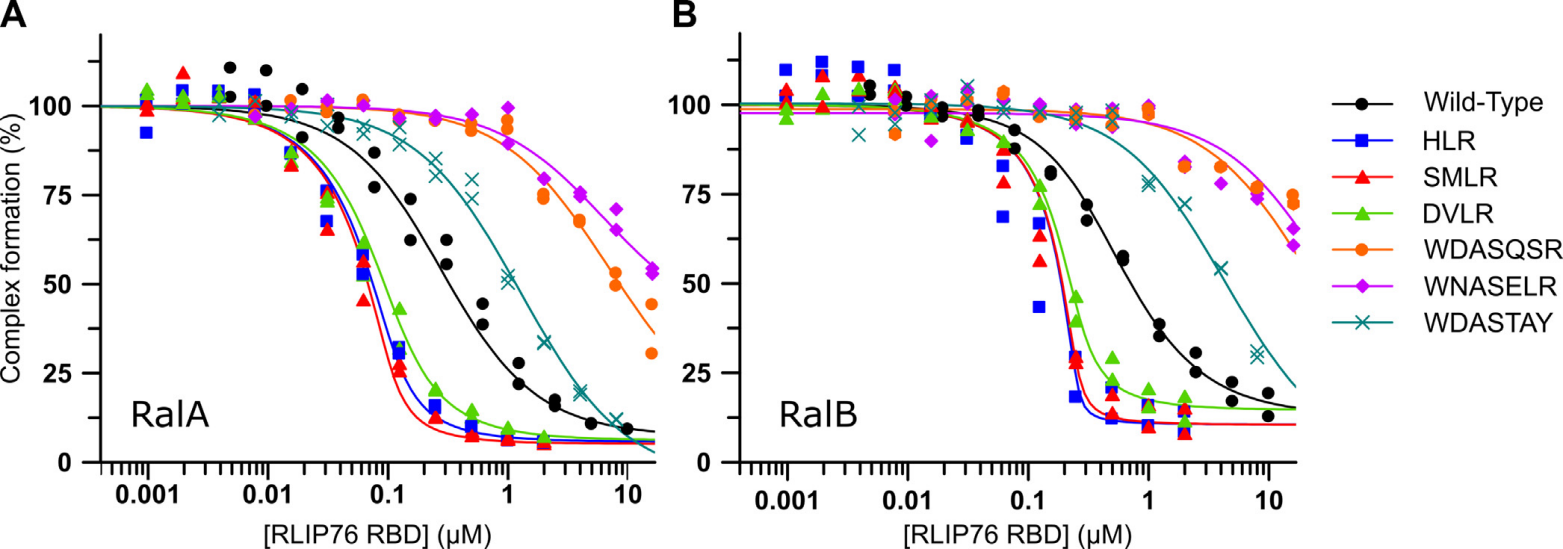
***In vitro* validation of CIS display hits.** Affinities were measured by competition SPAs. Mutant RBDs at the concentrations indicated were titrated into fixed concentrations of [^3^H]-GTP RalA (*A*) or [^3^H]-GTP RalB (*B*) and His-tagged RLIP76 RBD (wild-type) immobilized on SPA beads. Results from two independent experiments are shown, and data were fitted to the average result. Data and fits are displayed as a percentage of the maximum SPA signal measured for each condition. Data were fitted to a competitive binding isotherm describing a pure competition model to give apparent *K*_d_ (*K*_i_) values for the peptides as described previously (58).

Affinities of the sequences for Ral proteins were measured in competition SPAs (Fig. 2 and Table 1). Three out of six sequences tested bound more tightly than the wild-type RLIP76 RBD: all of these sequences were based on Cluster 1 (Fig. 1*C*) and the tightest binding sequences (HLR and SMLR) gave at least a 20-fold improvement in binding to both RalA and RalB. The sequences resembling Cluster 2 (Fig. 1*C*, WDASQSR, WNASELR and WDASTAY) all bound with lower affinity than the wild-type RLIP76 RBD. Competition SPA experiments however only assess binding at the same site as the immobilized effector, and it was possible that the sequences selected as Ral binders in the CIS display selections occupied an alternative site on the Ral proteins. To ensure this was not the case, the WDASQSR mutant was produced as a His-tagged construct and direct binding was assessed by SPA; however, the protein did not bind to RalA (Fig. S2) and circular dichroism (CD) experiments revealed that the coiled-coil structure of this mutant was disrupted (Fig. S3).

**Table 1:** Affinities of RLIP76 RBDs for RalA and RalB measured by competition SPA

Name	Sequence	*K*_d_ (nM)^a^	
		RalA·GTP	RalB·GTP
Wild-type	RLIP76 (393–422, C411S)-LSKEERLWEVQRILTALKRKLREA	96 ± 17	109 ± 16
HLR	....H.....L......R......	5 ± 3	1 ± 2
SMLR	....S.M...L......R......	3 ± 3	2 ± 2
DVLR	....D.V...L......R......	12 ± 4	7 ± 2
WDASQSR	...WD.A...SQ..S..R......	3020 ± 720	6970 ± 930
WNASELR	...WN.A...SE..L..R......	2750 ± 850	10,350 ± 2250
WDASTAY	...WD.A...ST..A..Y......	530 ± 70	1200 ± 140

a Standard error from curve fitting.

### Co-crystal structures of mutant RBD complexes

**Figure 3:**
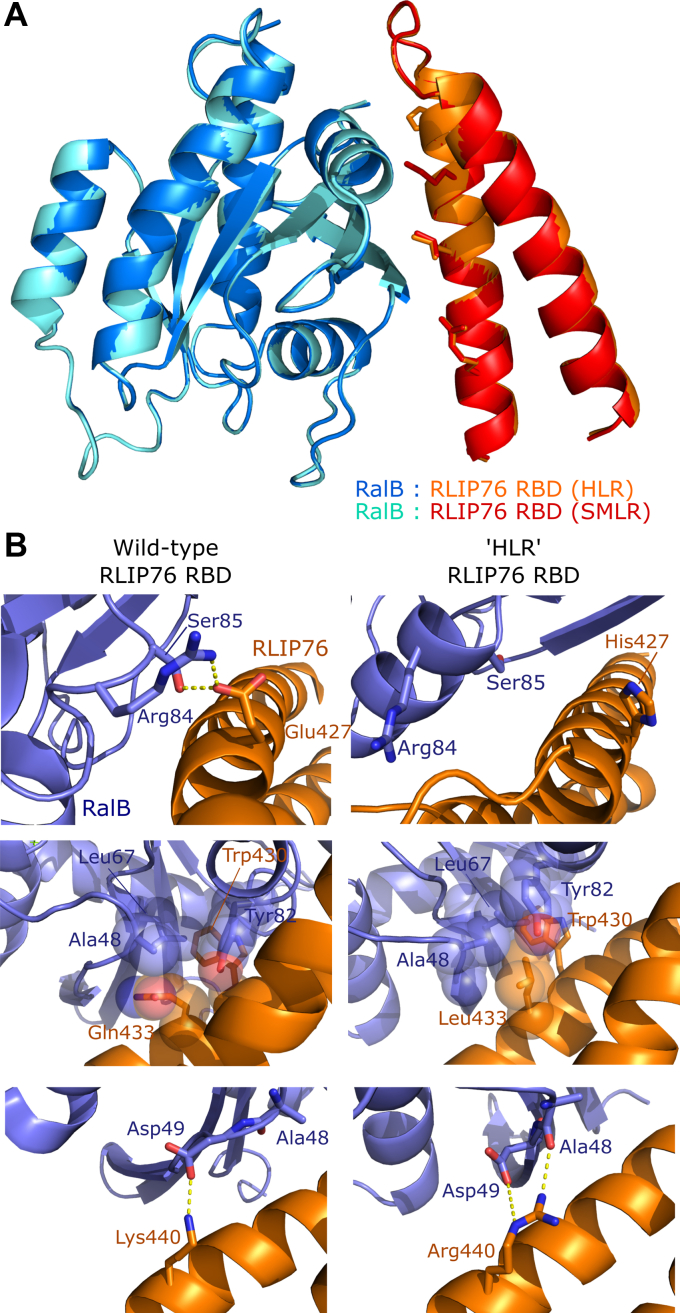
**Structures of the HLR and SMLR mutant RBDs in complex with RalB.***A*, an overlay of the structures of the HLR mutant (*orange*, PDB ID: 6ZQT) and SMLR mutant (*red*, PDB ID: 6ZRN) RLIP76 RBDs in complex with RalB·GMPPNP. *B*, zoomed views of the wild-type RBD/RalB complex (*left three panels*, PDB ID: 2KWI) and the HLR-mutant RBD complex (*right three panels*, PDB ID: 6ZQT), showing the interactions made by mutated residues. RalB is shown in *blue*, while the RLIP76 RBD is shown in *orange*.

To investigate the mechanism(s) driving the improved binding of the mutants to the Ral proteins, we set out to obtain structural data on the complexes. We obtained high-quality crystals for both the RalB/RLIP76 RBD HLR and SMLR mutant complexes, which diffracted to 1.5 Å resolution (Fig. 3*A* and Table 2). The two structures were highly similar with an RMSD less than 0.1 Å and all interactions formed with Ral proteins were identical, suggesting that the E427H/S and R429M mutations were not important for binding.

**Table 2:** Data collection and refinement statistics for RalB·GMPPNP in complex with the RLIP76 RBD HLR mutant (6ZQT) and SMLR mutant (6ZRN)

Protein complex	RalB·GMPPNP:RLIP76 RBD (HLR)^a^	RalB·GMPPNP:RLIP76 RBD (SMLR)^a^
PDB identifier	6ZQT	6ZRN
Resolution (Å)	50.4–1.51 (1.55–1.51)	65.8–1.48 (1.56–1.48)
Space group	P 1 2_1_ 1	P 1 2_1_ 1
Cell dimensions		
a,b,c (Å)	47.5, 77.4, 66.4	47.2, 77.5, 65.8
α,β,γ (°)	90, 90.3, 90	90, 90.1, 90
Total reflections	569,182 (24,662)	1,159,648 (176,059)
Redundancy	7.6 (4.5)	14.8 (15.4)
Completeness (%)	99.7 (98.7)	100.0 (100.0)
*I*/σ	18.3 (1.2)	8.0 (1.3)
Wilson B-factor (Å^2^)	24.5	19.3
**Refinement**		
R_work_/R_free_ (%)	18.9/22.1 (33.6/36.3)	20.1/23.9 (34.9/36.1)
No. of protein atoms	3686	3725
No. of ligand atoms	72	78
No. of water molecules	362	244
RMSD bond length (Å)	0.005	0.006
RMSD bond angles (°)	0.75	0.80
Ramachandran statistics		
In favored regions (%)	97.1	96.9
In allowed regions (%)	2.9	3.1
Outliers (%)	0	0
Mean B-factor (Å^2^)	34.5	30.8

a The numbers in parentheses represent values for the highest-resolution shell.

**Figure 4:**
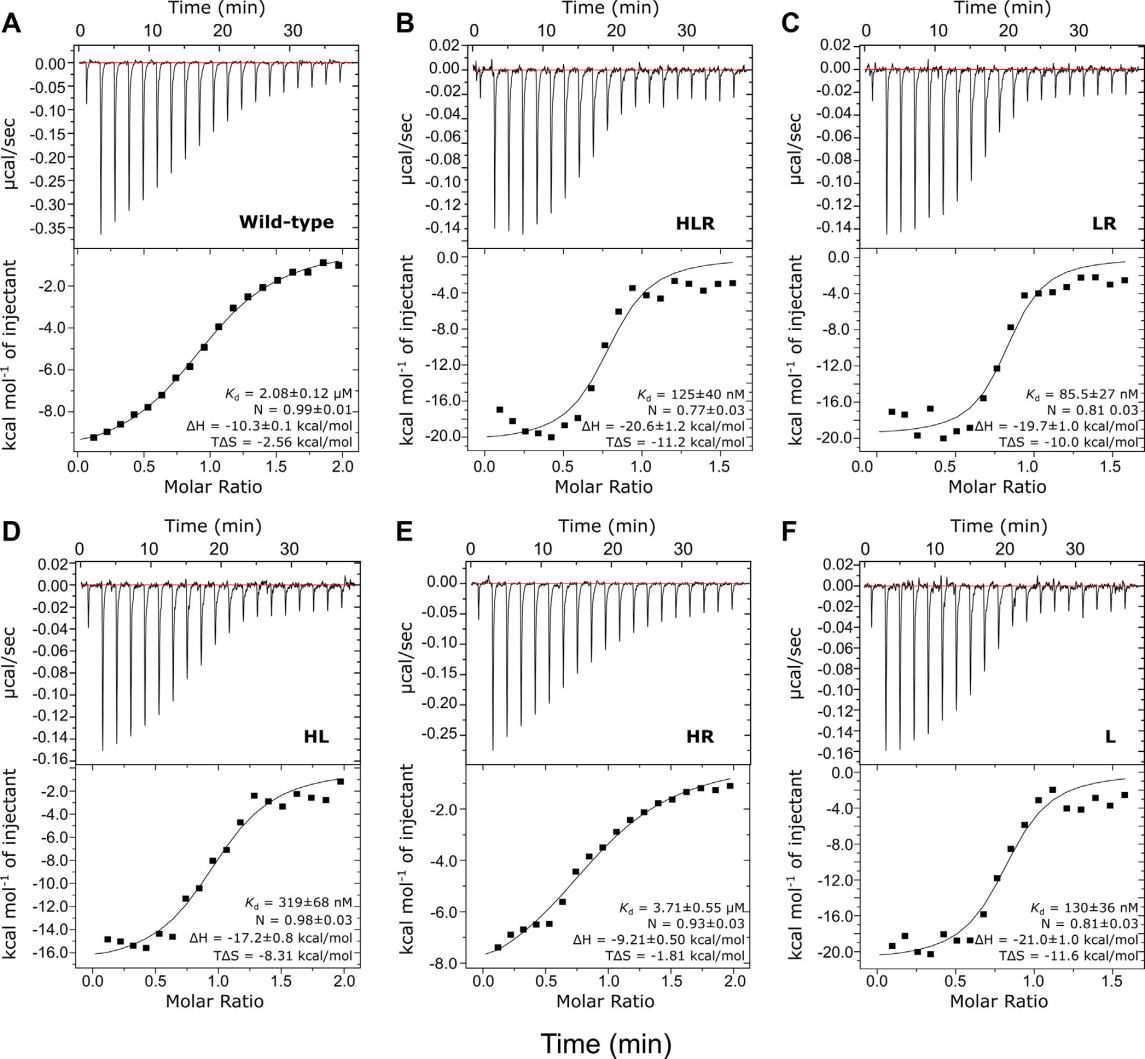
**Dissecting improved binding of the HLR mutant RBD to RalB.** Representative data from ITC experiments are shown for titrations of RalB·GMPPNP into the indicated RLIP76 RBD constructs. The parameters for the fit for these individual experiments are shown in each panel. For the average parameters obtained from two experiments, see Table 3. *A*, wild-type RLIP76 RBD, (*B*) HLR, (*C*) LR, (*D*) HL, (*E*) HR, (*F*) L mutant.

To delineate the thermodynamic contribution of the individual mutations in the HLR variant, constructs lacking each one of the changes were prepared and the binding of the HLR triple mutant and the three double mutants to RalB was assessed by isothermal titration calorimetry (ITC) (Fig. 4 and Table 3). The affinity of the wild-type RLIP76 RBD for RalB measured by ITC was 2.7 μM, in agreement with the previously reported value of 1.9 μM (34). The binding affinities measured by ITC differed from those measured by SPA competitions (approximately tenfold for the wild-type RBD); therefore, only comparisons of values within a given technique have been made.

**Table 3:** Binding parameters obtained from ITC for RLIP76 RBDs titrated into RalB

Name	Sequence	*K*_d_ (nM)^a^	N value^a^	ΔH (kcal/mol)^a^	TΔS (kcal/mol)^a^
Wild-type	RLIP76(393–422, C411S)-LSKEERLWEVQRILTALKRKLREA	2720 ± 640	1.00 ± 0.01	−10.5 ± 0.2	−2.89 ± 0.33
HLR	....H.....L......R......	96.2 ± 29.1	0.89 ± 0.12	−17.7 ± 2.9	−8.12 ± 3.08
LR	..........L......R......	132 ± 46	0.81 ± 0.00	−21.0 ± 1.3	−11.6 ± 1.5
HL	....H.....L.............	224 ± 94	0.83 ± 0.15	−19.5 ± 2.3	−10.3 ± 2.0
HR	....H............R......	4350 ± 630	0.99 ± 0.06	−9.05 ± 0.16	−1.72 ± 0.08
L	..........L.............	110 ± 19	0.75 ± 0.06	−22.4 ± 1.4	−12.9 ± 1.3

a Data reported are the mean values from two independent experiments ± one standard deviation.

As we have found previously, the interaction between RalB and the wild-type RLIP76 RBD was driven by a favorable enthalpic term (ΔH), which outweighs the entropic cost of binding (ΔS) (34). The HLR mutant increased the binding to RalB 28-fold, which is broadly similar to the improvements seen by SPA. The binding of the three double mutants revealed that the improvement in affinity was only observed when the Q433L mutation was present. A variant harboring the single Q433L mutation was produced and bound with a similar affinity to the HLR triple mutant, indicating that the Q433L mutation alone was sufficient to increase the affinity for RalB by 25-fold.

The mutant RBD structures show that the side chain of Leu433 contributes to a hydrophobic network of interactions involving several Ral residues (Ala48, Leu67, Tyr82) and Trp430 of RLIP76 (Fig. 3*B*, middle panels). In the wild-type RBD complex, Gln433 points out to the solvent, leaving a space at the edge of the hydrophobic pocket where Trp430 is buried in an interaction known to be essential for the binding affinity. When the Gln is replaced by Leu, the side chain shifts toward the pocket and fills this space.

Despite the obvious hydrophobic interactions that Leu433 makes in the complex, the ITC data showed that when RalB binds the Q433L mutant, the improved affinity compared with the wild-type RBD is due to a twofold increase in the favorable enthalpic contribution. Furthermore, the entropic cost of binding for the Q433L mutant was three- to fourfold greater than that for the wild-type RBD, even though hydrophobic-driven interactions are usually characterized by a favorable entropic change. This suggests that the Q433L mutation in the RBD has a more nuanced effect on binding than simply contributing to the hydrophobic pocket around Trp430, perhaps by altering the presentation of other RBD residues.

Lys440 forms a hydrogen bond with RalB Asp49 in the wild-type complex (Fig. 3*B*, bottom panels) and this interaction has been shown to be critical for binding to Ral, as alanine replacement reduced the affinity tenfold (36). Replacement with arginine in the HLR and SMLR mutants allows this hydrogen bond to be maintained while an additional hydrogen bond with the backbone carbonyl of RalB Ala48 is gained. However, the K440R mutation only exerts a minimal improvement in binding of the RBD to Ral proteins, as evidenced by comparison of the E427H/Q433L (HL) double mutant with the E427H/Q433L/K440R (HLR) triple mutant, which bind with similar affinities (Fig. 4 and Table 3).

The replacement of Glu427 with histidine or serine breaks a salt bridge formed with RalB without forming any new interactions (Fig. 3*B*, top panels) and ITC data demonstrated that this substitution did not improve binding to Ral proteins (Fig. 4 and Table 3). Analysis of the sequences in Cluster 3 showed that the Q433L/K440R substitutions appeared together with high frequency, while position 427 was replaced by a range of amino acids. These observations, taken together, suggest that the conserved substitutions Q433L and K440R are the driving force for higher-affinity binding.

### Second-generation stapled peptides

We aimed to use the insights gained from the CIS display selections to improve the affinity of the first-generation stapled peptides targeting the Ral GTPases. In previous work we investigated a range of staple positions for peptides comprising residues 423 to 446 of the RLIP76 RBD (the α2 helix) and found that a staple bridging residues 424 to 428 produced the tightest binder, termed SP1 (34). This lead peptide bound to RalA and RalB with *K*_d_ values of 14 and 5 μM, respectively, and showed some selectivity for the Ral GTPases, binding more weakly to the related GTPase R-Ras with a *K*_d_ of 30 μM. Using a selection of the top hits identified in the CIS selection, we generated a series of peptides based on residues 423 to 446 of the RLIP76 RBD with the same staple position as the lead peptide (SP1).

**Figure 5:**
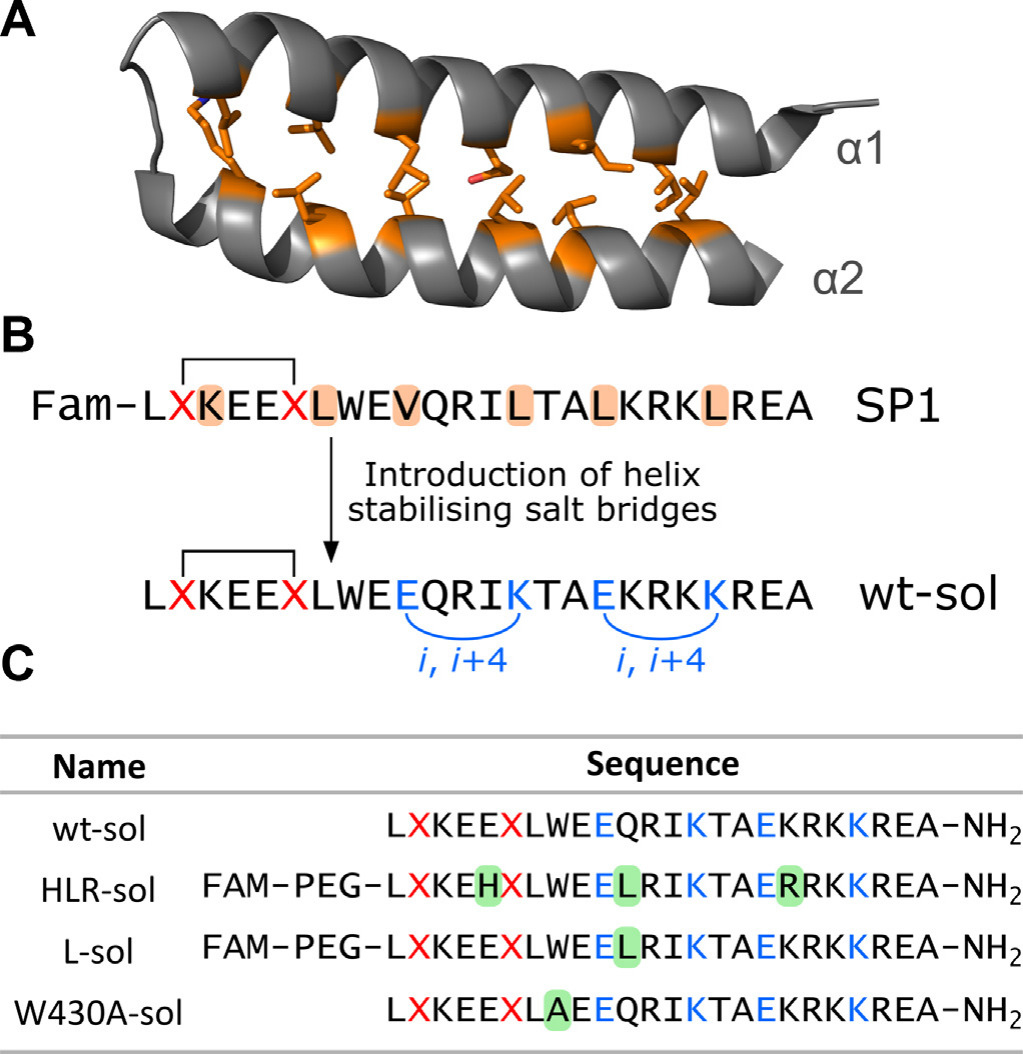
**Improving the solubility profile of stapled peptides based on RLIP76.***A*, the RLIP76 RBD structure (PDB ID: 2KWI) shows that the RLIP76 RBD coiled-coil is held together by hydrophobic residues at the helix interface (shown as *orange sticks*). *B*, modifications made to the lead stapled peptide (SP1) to improve solubility and stabilize the helical structure. Residues shaded *orange* in the SP1 sequence match those colored *orange* in the α2 helix in A. *C*, soluble peptide sequences were produced synthetically. Solubilizing salt bridges are shown in *blue*, and sequence changes from the “wild-type” (wt-sol) sequence are highlighted in *green boxes*. FAM, 5-carboxyfluorescein; PEG, polyethylene glycol linker, amino-4,7-dioxanonanoic acid; X, position of stapled residues.

Peptides corresponding to the wild-type α2 sequence (SP1) and those with the HLR sequence were compared for their binding to a panel of small GTPases using fluorescence polarization. This investigation into the specificity of the lead peptide, SP1, revealed issues with poor solubility and nonspecific binding to several other small GTPases (see Fig. S4 and Table S1), particularly RhoA: this nonspecific binding was tighter in the HLR peptides. The peptides have a hydrophobic back face with exposed residues that would be buried within the coiled-coil interface in the RBD and the all-hydrocarbon staple further increases this hydrophobic surface. We reasoned that if the solubility profile of the peptides could be improved, this could increase the specificity. We hypothesized that the hydrophobic residues on the back face of the peptide could be replaced without negatively impacting binding affinity for Ral proteins and chose to substitute these with charged residues (glutamate and lysine), which would decrease hydrophobicity and simultaneously create two *i*, *i* + 4 salt bridges to help stabilize the helical structure of the peptides (Fig. 5) (39). The resulting peptides were readily soluble in aqueous solution.

**Figure 6:**
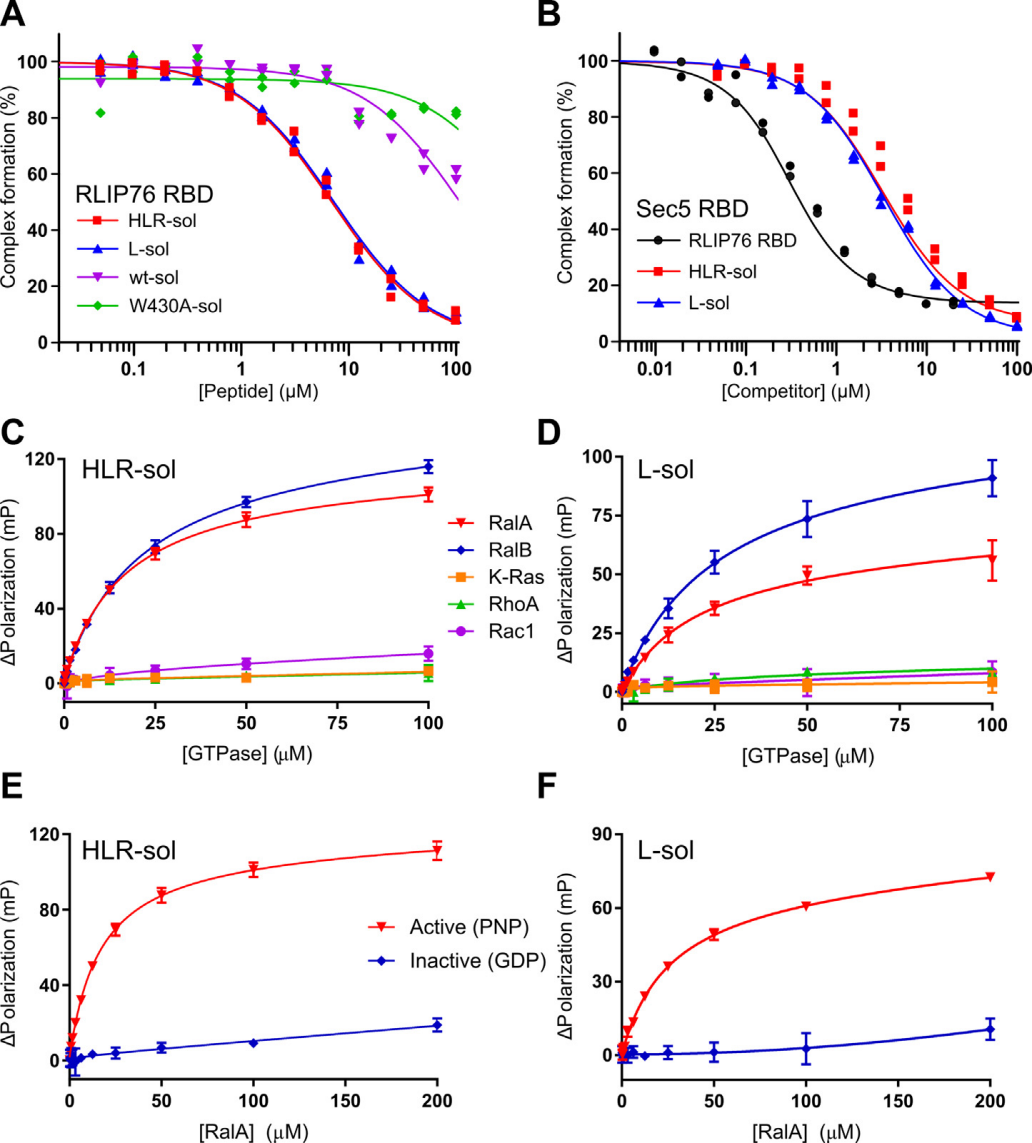
**Binding of soluble peptides to Rals and related small GTPases.***A*, peptides at the concentrations indicated were titrated into fixed concentrations of [^3^H]-GTP RalA and His-tagged RLIP76 RBD (wild-type) immobilized on SPA beads. Data and fits were produced as described for Figure 2. *K*_d_ = HLR-sol, 2.97 ± 0.29 μM; L-sol, 3.09 ± 0.32 μM; wt-sol, 48.6 ± 7.7 μM; W430A-sol, > 100 μM. *B*, peptides and the RLIP76 RBD at the concentrations indicated were titrated into fixed concentrations of [^3^H]-GTP RalA and GST-tagged Sec5 RBD immobilized on SPA beads. Data and fits were produced as described for Figure 2: *K*_d_ = RLIP76 RBD, 115 ± 30 nM; HLR-sol, 1.74 ± 0.15 μM; L-sol, 1.81 ± 0.14 μM. *C*–*D*, FP data for direct binding of 20 nM FAM-labeled HLR-sol (*C*) and L-sol (*D*) to varying concentrations of indicated GMPPNP-bound small GTPases. n = 3, except for K-Ras where n = 2. HLR-sol: *K*_d_ = 16.6 ± 1.3 μM, RalA; 21.1 ± 1.8 μM, RalB. L-sol: *K*_d_ = 19.8 ± 3.9 μM, RalA; 24.4 ± 5.0 μM, RalB. No *K*_d_ values could be estimated for K-Ras, RhoA, or Rac1 as binding was too weak. *E*–*F*, FP data for direct binding of 20 nM FAM-labeled HLR-sol (*E*) and L-sol (*F*) to varying concentrations of RalA·GMPPNP and RalA·GDP. *K*_d_ values for RalA·GMPPNP are as in *D*–*E*. No *K*_d_ values could be estimated for RalA·GDP as binding was too weak. n = 3.

The affinities of the soluble peptides for RalA were defined in competition SPAs by measuring their ability to compete with the RLIP76 RBD (Fig. 6*A* and Table 4). The peptides containing single or triple mutations identified from the CIS display selection, modified to contain the solubilizing residues (designated HLR-sol and L-sol), both bound with a *K*_d_ of approximately 3 μM, which was a 16-fold improvement on the “wild-type” sequence containing the same solubilizing mutations (wt-sol, *K*_d_ RalA = 49 μM). This demonstrates that the sequences identified using the RLIP76 RBD could be directly translated to produce peptide sequences with improved affinity for the Ral GTPases. Meanwhile, a peptide in which the critical tryptophan residue had been changed to alanine (W430A-sol), designed as a negative control peptide, showed very weak binding to RalA.

**Table 4:** Affinity measurements for second-generation peptides binding to a panel of small GTPases

GTPase	Assay	*K*_d_ (μM)^a^	
		HLR-sol	L-sol
RalA·GTP	Competition SPA (RLIP76)	2.97 ± 0.29	3.09 ± 0.32
	Competition SPA (Sec5)	1.74 ± 0.15	1.81 ± 0.14
RalB·GMPPNP	FP	16.6 ± 1.3	19.8 ± 3.9
RalA·GDP	FP	NB	NB
RalB·GMPPNP	FP	21.1 ± 1.8	24.4 ± 5.0
K-Ras·GMPPNP	FP	NB	NB
RhoA·GMPPNP	FP	NB	NB
Rac1·GMPPNP	FP	NB	NB
		**wt-sol**	**W430A-sol**
RalA·GTP	Competition SPA (RLIP76)	48.6 ± 7.7	>100

NB, no binding.

a Standard errors from curve fitting.

These data demonstrate that the HLR-sol and L-sol peptides are competitive with the RLIP76 RBD for binding to Ral proteins. We predicted that the peptides would also be able to inhibit other Ral–effector interactions; therefore, we tested the peptides in competition with the Sec5 RBD and found that they fully competed with Sec5 for binding to RalA (Fig. 6*B* and Table 4). The *K*_d_ values of 1.7 and 1.8 μM for HLR-sol and L-sol binding to RalA, respectively, agree with those measured by competition with the RLIP76 RBD.

Direct-binding fluorescence polarization assays were then used to assess selectivity of the peptides for Ral proteins over other small GTPases (Fig. 6, *C*–*D*, Table 4). K-Ras was chosen as the Ras proteins are closely related to the Ral proteins, sharing more than 50% sequence identity, while Rac1 and RhoA were chosen as representatives of the Rho family of small GTPases. The HLR-sol and L-sol peptides bound to RalA and RalB with *K*_d_ values between 17 and 24 μM and showed no off-target binding to the other GTPases tested, demonstrating that the peptide selectivity is dramatically improved with the inclusion of the solubilizing mutations. The nonsolubilized peptides bound to the other GTPases with affinities that were similar to those of the Ral proteins. The solubilized peptides showed no binding to the no-Ral GTPases at concentrations up to 100 μM, indicating that any binding to the other GTPases was at least an order of magnitude lower in affinity than that of the Ral proteins.

As our ultimate aim was to disrupt Ral–effector interactions for therapeutic purposes, it was highly desirable that the peptides were also selective for the active form of the Ral GTPases. The peptides demonstrated very little binding to RalA·GDP and are therefore selective for the active, GTP-bound form of Ral (Fig. 6, *E*–*F*, Table 4). These data also suggest that the peptides are binding at the nucleotide-sensitive switch regions, which are the known binding sites for most Ral effectors and regulatory proteins. This is in agreement with the peptides’ ability to compete with effector proteins, which also bind to the switch regions.

### Mapping the peptide binding site by NMR

**Figure 7:**
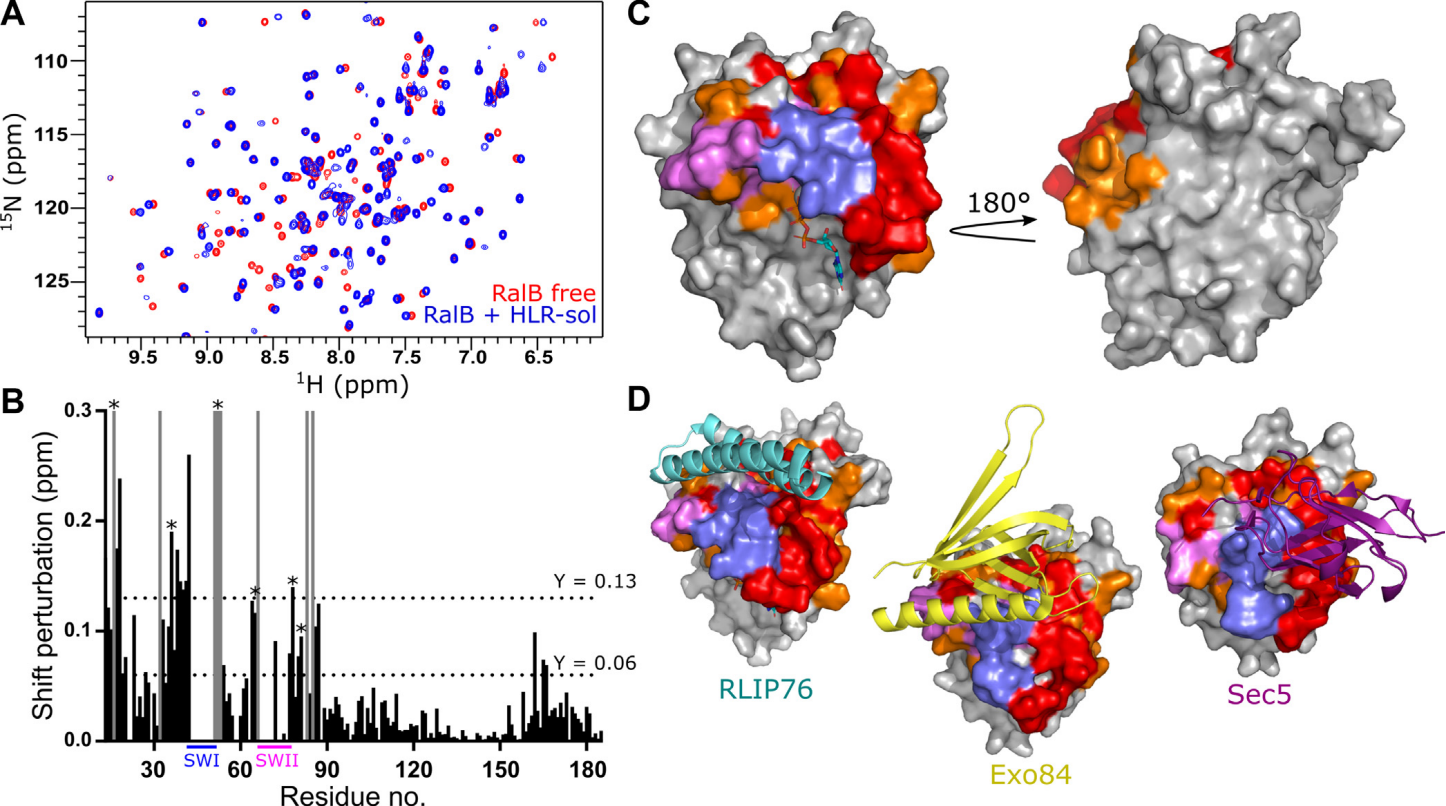
**Titration of HLR-sol into**^**15**^**N-labeled RalB.***A*, HSQC spectra of ^15^N-labeled RalB alone (*red*) and after the addition of two equivalents of HLR-sol peptide (*blue*). *B*, chemical shift perturbation for backbone amides of RalB after addition of two equivalents of HLR-sol peptide. Peaks that have shifted too far to be reliably assigned have been given a chemical shift perturbation of 0.3 ppm and are shown in gray. Some switch region residues are missing from the free RalB spectrum owing to their conformational flexibility; therefore, shift distances cannot be assigned and no bar is shown. The average shift change (0.06) and the average plus one standard deviation (0.13) are marked by *dotted lines*. The location of switch I residues (SWI, 41–51) is indicated in *blue*, and switch II residues (SWII, 69–81) are indicated in *magenta*. Shifts marked with a *star* (∗) indicate those that are involved in binding to Exo84 that shift on peptide binding. *C*, residues with the largest chemical shift distances mapped onto the surface of RalB (PDB: 2KWI). Shifts greater than 0.13 ppm are colored *red* and those greater than 0.06 ppm are colored *orange*. Switch I residues are colored *blue*, and switch II residues are colored *magenta*. *D*, structures of Ral with RLIP76 RBD (*cyan*, PDB ID: 2KWI), Exo84 RBD (*yellow*, PDB ID: 1ZC3), and Sec5 RBD (*purple*, PDB ID: 1UAD), with RalB colored as in *C*.

The binding site of the HLR-sol peptide was next investigated using chemical shift mapping by NMR spectroscopy. HSQC spectra of ^15^N-labeled RalB alone and in the presence of increasing amounts of unlabeled peptide were recorded (Fig. 7*A*). Backbone amide peaks that shift position during the titration correspond to residues that are perturbed on peptide binding. Most of these will be within or proximal to the peptide binding site, although some can report on small conformational differences in the protein on peptide binding. The distances that each residue shifted are shown plotted in Figure 7*B*. During the titration, some peaks disappeared or moved too far to be reliably assigned and have been given an arbitrary value of 0.3 ppm. Residues whose shifts were perturbed were mapped onto RalB (Fig. 7*C*). These are clearly localized on one surface of the protein. Although not all the residues within the switch regions were visible in the free RalB spectra owing to their conformational exchange, several surrounding residues were shifted, suggesting that the peptide is binding at the switches in accordance with previous data. Residues Tyr51 and Arg52, just C-terminal to switch 1, disappeared during the titration: these residues are therefore likely involved in peptide binding and have been shown previously to be involved in Ral interactions with effector proteins (35, 40). An overlay of the peptide binding site obtained by chemical shift mapping with known Ral–effector complexes suggests that there is a high degree of overlap with the effector-binding interfaces (Fig. 7*D*).

### Disruption of Ral–effector complexes in mammalian cell lysates

**Figure 8:**
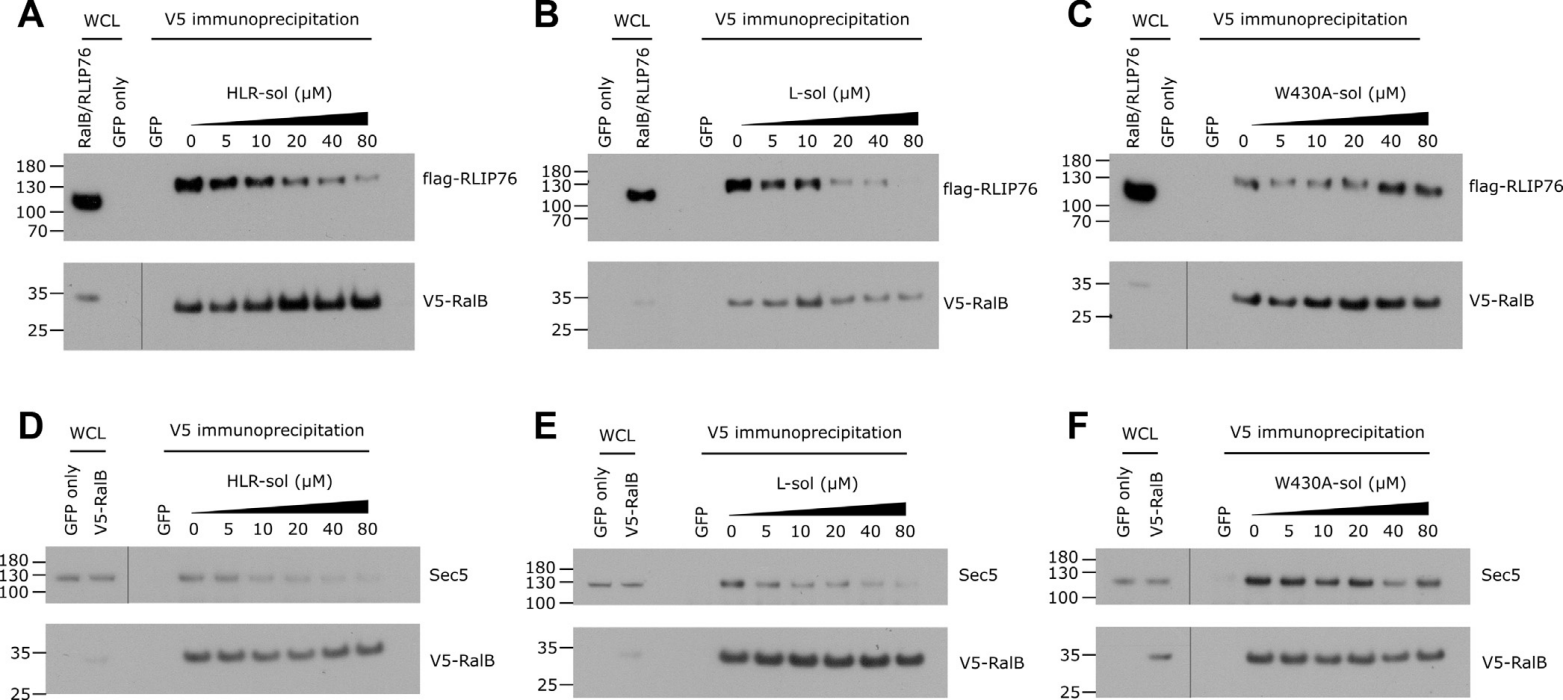
**Disruption of Ral–effector complexes by second-generation stapled peptides in a mammalian cell lysate.***A*–*C*, the indicated concentrations of HLR-sol (*A*), L-sol (*B*), and W430A-sol (*C*) stapled peptides were added to HEK293T cell lysates transfected with GFP only or V5-tagged RalB Q72L and flag-tagged RLIP76 24 h prior to lysis. Beads coated with an anti-V5 antibody were added to the lysate mixture to precipitate RalB and any bound proteins. The presence of RalB and bound RLIP76 was assessed by probing with antiflag (RLIP76) and anti-V5 (RalB). WCL, whole cell lysate. *D*–*F*, coimmunoprecipitations were performed without RLIP76-flag transfection as above in the presence of increasing concentrations HLR-sol (*D*), L-sol (*E*), and W430A-sol (*F*) stapled peptides. The presence of RalB and bound Sec5 was assessed by probing with anti-Sec5 and anti-V5 (RalB).

Competition SPAs (Fig. 6, *A*–*B*) demonstrated that the optimized peptides were able to disrupt Ral effector complexes *in vitro*; however, we also wanted to test the selectivity of the peptides for Ral in a complex cell mixture. We therefore examined whether the peptides could disrupt Ral–effector complexes in a mammalian cell lysate. HEK293T cells were transfected with V5-tagged, constitutively active (Q72L), RalB alone, or with flag-tagged RLIP76. Peptides were incubated in the cell lysate at the concentrations indicated prior to RalB immunoprecipitation *via* its V5 tag. The presence of bound effector proteins, endogenous Sec5 and flag-tagged RLIP76, was assessed by western blotting (Fig. 8). The HLR-sol and L-sol peptides were able to inhibit the interactions between Ral and the effector proteins in a dose-dependent manner, whereas the negative control peptide with a W430A mutation did not inhibit complex formation.

### Assessment of peptide helicity by circular dichroism

**Figure 9:**
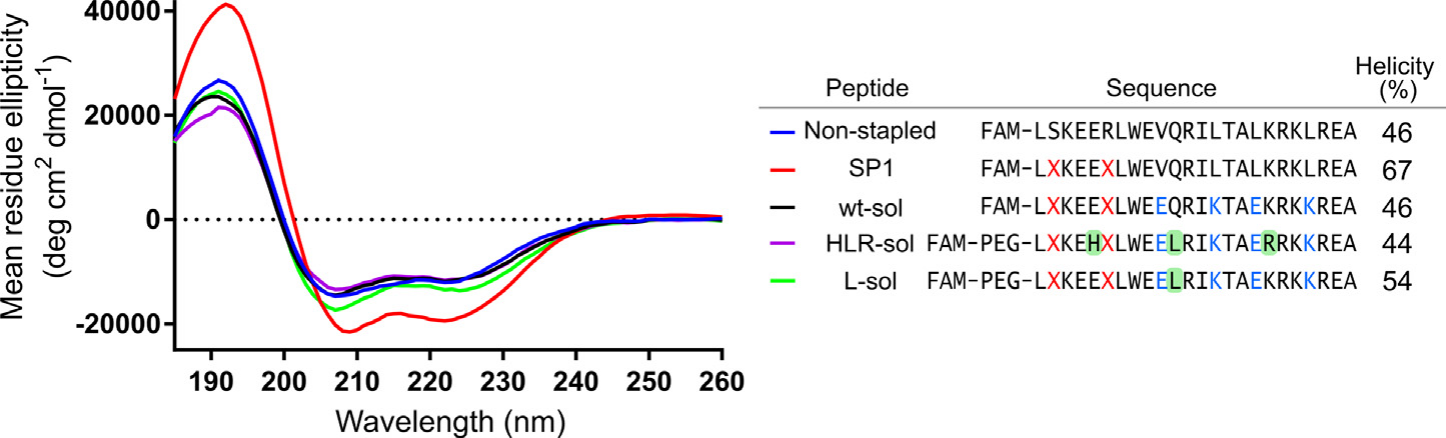
**CD spectra of stapled peptides.** CD spectra of the peptides were measured over the wavelengths 185 to 260 nm with a peptide concentration of 0.2 mg/ml. The reported helicity (%) was estimated by the CDSSTR method and reference set 3 using Dichroweb (59, 60, 61). Residues forming the staple are shown as *red* Xs, solubilizing mutations are shown in *blue*, and residues varying from the wt-sol template in HLR-sol and L-sol are shown in *green squares*.

CD was used to assess the helicity of the original (SP1) peptide (34) and the second-generation peptides with solubilizing mutations (Fig. 9). All peptides had some helical character, as demonstrated by the double minima at 208 and 222 nm. Addition of the solubilizing salt bridges to the wild-type peptide reduced the overall helicity to the same level as the unstapled control peptide. Similarly, the HLR-sol peptide with solubilizing salt bridges was less than 50% helical. The L-sol peptide was slightly more helical that the HLR-sol peptide, implying that replacement of Glu with His or Lys with Arg reduced the helicity of the HLR-sol peptide. The helical propensity of Glu is higher than that of His, while the propensity of Lys and Arg is similar (41), suggesting that it is the His substitution that affects helicity most. Nevertheless, the addition of two salt bridges, which replaced hydrophobic residues with charged sidechains, reduced the overall helicity compared with the original sequence. It is likely that the original hydrophobic residues could form hydrophobic interactions with multiple surrounding residues, which could stabilize a helical structure (42).

Several studies have found that target affinity improves with increased peptide helicity (43, 44), raising the possibility that these peptides would have improved binding to Ral proteins if their helicity could be improved, *e.g.*, by altering the solubilizing residues, incorporation of alpha-substituted amino acids or inclusion of a second staple.

### Contributions of residues in helix α1 of the RLIP76 RBD

**Figure 10:**
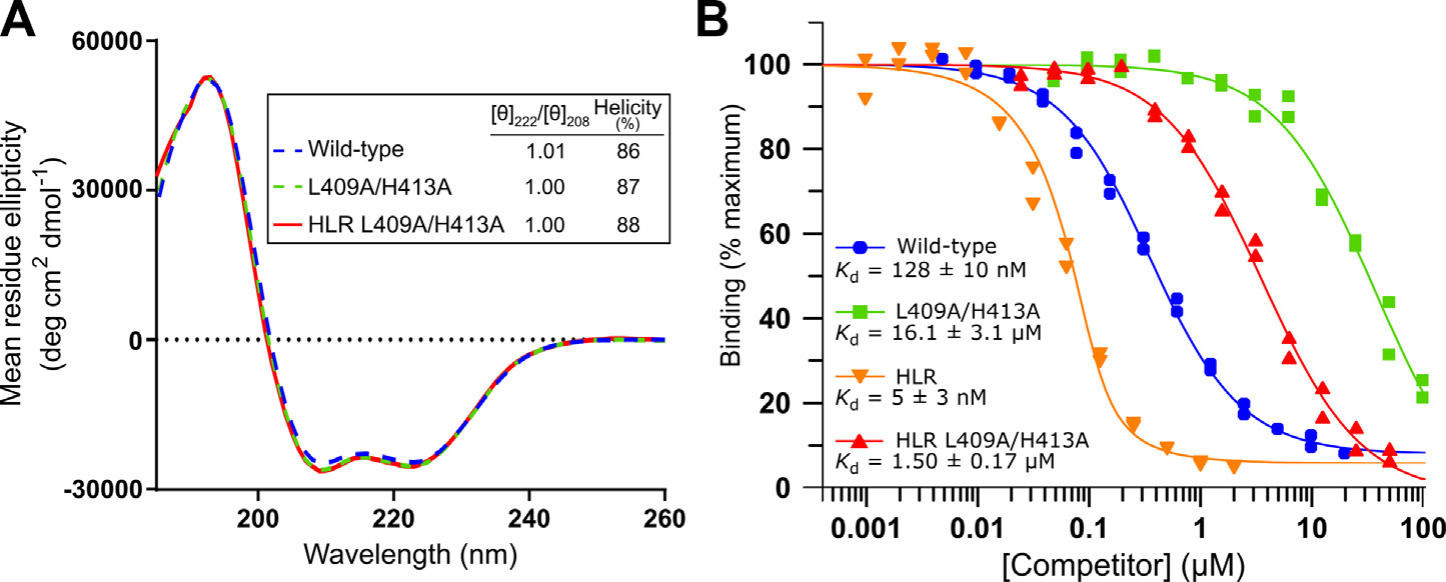
**Binding of RLIP76 RBDs with key α1 helix residues mutated to alanine.***A*, CD spectra of the wild-type RLIP76 RBD and L409A/H413A RBD mutants were measured over the wavelengths 185 to 260 nm with a protein concentration of 0.2 mg/ml. The calculated helicities and ratios of the mean residue ellipticities (θ) at 222 and 208 nm ([θ]_222_/[θ]_208_) are shown in the inset. *B*, Binding of the L409A/H413A mutant RBDs to RalA was measured by competition SPA. RBDs at the indicated concentrations were titrated into a fixed concentration of [^3^H]-GTP·RalA and His-tagged wild-type RLIP76 RBD immobilized on SPA beads. Data and fits were produced as described for Figure 2: *K*_d_ = wild-type, 128 ± 10 nM; L409A/H413A, 16.1 ± 3.1 μM; HLR, 5 ± 3 nM; HLR L409A/H413A, 1.50 ± 0.17 μM.

In the context of the RLIP76 RBD, which is ∼90% helical (Fig. S3), the HLR and L sequences bind tightly to Ral proteins. It is known that within the α1 helix of the RLIP76 RBD, which is not present in the peptides, residues Leu409 and His413 are involved in the interaction with Ral proteins. When these residues were previously mutated individually to alanine in the RLIP76 RBD, the binding to Ral proteins was significantly reduced (36). These key contacts were therefore both mutated to alanine, removing all binding contributions from the α1 helix within the RLIP76 RBD coiled-coil but preserving the interactions of the α2 helix. The L409A/H413A mutations were also made in the context of the HLR (E427H/Q433L/K440R) RBD mutant and CD was used to assess the helicity and coiled-coil structure of the constructs (Fig. 10*A*). Coiled-coil content can be estimated from CD data using the [θ]_222_/[θ]_208_ ratio, where [θ] is mean residue ellipticity at 222 and 208 nm, respectively: coiled-coils give values of approximately 1.0 while single α-helices give values closer to 0.8 (45, 46). The helicity was not affected by the introduction of the L409A/H413A mutations and the [θ]_222_/[θ]_208_ ratios confirmed that the coiled-coil content was also unaffected. The affinities of the L409A/H413A mutated RBD constructs for RalA were then measured by competition SPA (Fig. 10*B*). Comparison of the wild-type RBD and the L409A/H413A mutant showed that removal of the interactions made by Leu409 and His413 reduced the affinity ∼125-fold. Similarly, with the HLR mutations present, the affinity was reduced ∼300-fold, indicating that the contribution of the Leu409 and His413 sidechains to the RBD interaction is significant.

## Discussion

In this work, we successfully employed a CIS display maturation selection to identify sequence changes in the RLIP76 RBD that improve affinity for Ral proteins. In particular, the consensus sequence E427X/Q433L/K440R (Cluster 3, Fig. 1*A*), where X denotes a range of amino acids, produced sequences with the greatest improvement in affinity for Ral proteins. The tightest binding sequences, termed HLR (E427H/Q433L/K440R) and SMLR (E427S/L429M/Q433L/K440R), displayed affinities of 5 and 3 nM for RalA, respectively, a vast improvement on the wild-type RBD affinity of around 100 nM. ITC revealed that the Q433L mutation was the key driver for improved affinity, resulting in a ∼20 to 30-fold improvement in affinity compared with the wild-type RBD. Crystal structures of the HLR and SMLR mutant RBDs in complex with RalB revealed that this leucine side chain is able to sustain a network of hydrophobic interactions involving Ral and RBD residues.

We sought to apply these changes to our lead stapled peptide based on the α2 helix (residues 423–446) of the RLIP76 RBD (SP1), which we previously showed could bind to Ral proteins and inhibit RalB-mediated autophagy. However, further investigations into this peptide led us to uncover issues with nonspecific binding, confounding binding data generated previously. The lead peptide, SP1, was relatively hydrophobic and poorly soluble; therefore, we sought to improve the solubility of the peptides by the addition of salt bridges that could stabilize their helical structure. The addition of two salt bridges replacing hydrophobic residues on the back face of the peptide resulted in soluble peptides that no longer bound nonspecifically to other GTPases. The measured affinity of the soluble template (wt-sol, *K*_d_ RalA ≈50 μM) was, however, much weaker than our previous lead peptide, suggesting that the *K*_d_ of the lead peptide was at least partly a result of nonspecific effects, as equal and even greater affinities were measured for other small GTPases. Additionally, the SP1 lead peptide displayed an apparent *K*_d_ for RalB that was fivefold tighter than for RalA, despite the RLIP76 RBD having the same affinity for both Ral proteins. This suggests that the SP1 peptide either bound at a site differing from the RLIP76 RBD binding regions that are identical between RalA and RalB or was able to contact an additional site on RalB.

Recently, it has been found that several highly cited peptides suffer similar issues with nonspecific binding, including the hydrocarbon-stapled peptide targeting the K-Ras/SOS1 interaction (SAH-SOS1_A_), identified by Walensky and colleagues (47, 48). Fluorescence polarization (FP) assays were shown to be particularly susceptible to these misleading results, with nonspecific binding observed for a range of nontarget proteins and to plate surfaces, demonstrating the importance of validating peptide binding through orthogonal assays. We established that the nonspecific binding of our SP1 peptide in FP assays was due to a hydrophobic patch on the back face of the peptide, comprising residues that would normally be embedded in the coiled-coil interface and exacerbated by the hydrocarbon staple. Our findings join other examples demonstrating that care must be taken when using peptide sequences that are not normally solvent-exposed in the parent protein structure. However, we also demonstrate here that careful modifications, such as the introduction of charged residues, can resolve these issues, when the problematic regions are identified through the analysis of known structures.

When the sequence substitutions identified by CIS display were included in the soluble peptide template to produce the HLR-sol and L-sol peptides, the affinity improved to a similar extent as in the RLIP76 RBD. Both peptides bound with a *K*_d_ of 3 μM measured by competition SPA, an improvement of around 16-fold compared with the wild-type template, while the same substitutions in the RBD (HLR mutant) improved affinity ∼20-fold. The optimized peptides were selective for the active form of Ral proteins, with minimal binding observed for the GDP-bound form, and were highly selective for Ral proteins over a panel of small GTPases. NMR titrations of the HLR-sol peptides also confirmed that the peptide was bound at the effector-binding surface on RalB, while the competition SPA and coimmunoprecipitation experiments revealed that the peptides were competitive with binding of multiple Ral effectors, RLIP76 and Sec5.

We also attempted to assess whether the peptide could disrupt RalB/Exo84 complex formation in coimmunoprecipitation experiments; however, no validated commercial Exo84 antibodies were compatible with this methodology. Jin *et al.* (40) have previously identified the RalA residues involved in binding to Exo84 and in fact all of these residues that were observable in our NMR mapping experiments. Lys16, Tyr36, Arg52, Asp65, Ile78 and Asn81 experienced significant chemical shift perturbations on titration of the peptide, HLR-sol, into ^15^N-labeled RalB (Fig. 7*B*). This suggests that the HLR-sol peptide is utilizing many of the same residues as Exo84 for binding and is therefore likely to also compete with this effector protein. There are also likely to be more shared residues in the switch regions that were missing from our NMR spectra due to their conformational flexibility.

**Figure 11:**
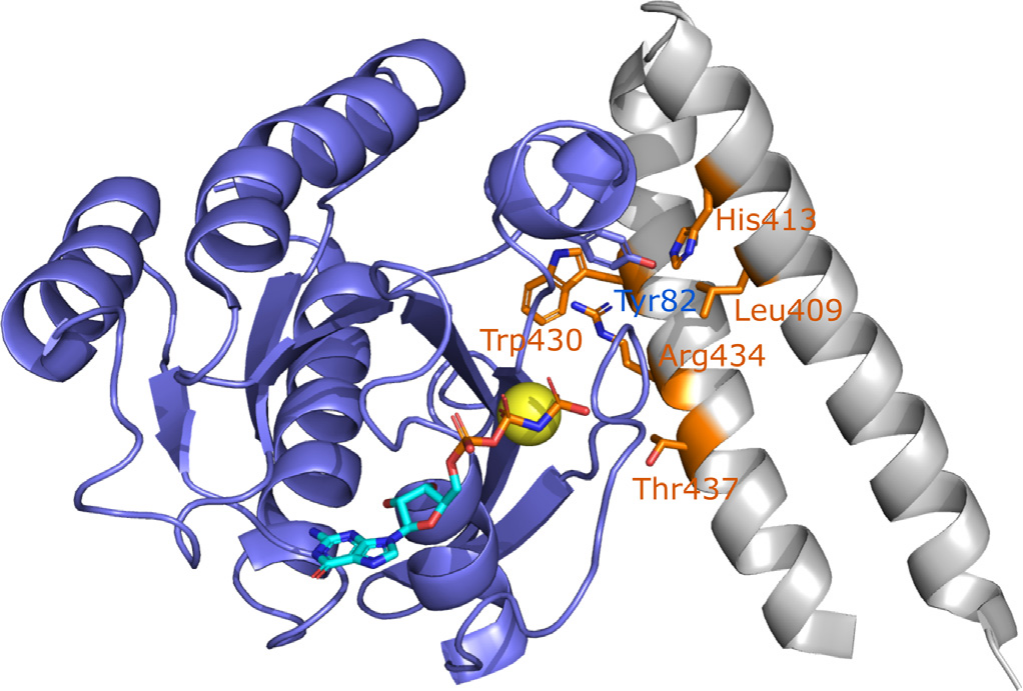
**RLIP76 RBD helix 1 contacts.** The NMR structure of RalB·GMPPNP in complex with the RLIP76 RBD (PDB ID: 2KWI) is shown, with RalB colored *blue* and the RLIP76 RBD colored *gray*. Residues within the RLIP76 RBD that, upon mutation to alanine, reduce binding to Ral more than tenfold are shown in *sticks* and are colored in *orange* (36). In the α1 helix, these residues are Leu409 and His413. Tyr82 on Ral that has been exploited for covalent inhibition is shown as *sticks* and forms a hydrogen bond with His413 of the RLIP76 RBD.

Inclusion of the salt bridges to improve solubility lowered the helical content of the peptides, presumably owing to disruption of a hydrophobic network of interactions on the back face of the peptide. To assess whether the lower helicity was impacting the binding affinity, a model RLIP76 RBD construct in which the binding contacts in the α1 helix were removed (L409A/H413A, Fig. 11) was used to estimate the maximal affinity of a peptide sequence based on the α2 helix alone. Assuming that Leu409 and His413 are the only significant contact points on helix 1, the HLR L409A/H413A variant RLIP76 RBD is a mimic for an HLR peptide with a stabilized helix. It is therefore informative to compare the *K*_d_ of the HLR L409A/H413A RBD (1.5 μM) with that of the HLR-sol stapled peptide (3 μM, Fig. 6*A*). These data suggest that increasing the helicity of the peptide would only improve affinity for Ral proteins by around twofold; therefore, no further attempts to improve peptide helicity were made. Predictably, it is likely that Leu409 and His413 make the crucial contacts that contribute to the increased affinity of the RBD over the peptides.

To improve the affinity of the peptides, the sequence could be extended in order to include the critical binding contacts in the α1 helix, Leu409, and His413. To this end, it would be highly desirable to have a reliable method to stabilize coiled-coil peptides in addition to single helices, especially given that a large number of GTPase/effector interactions are mediated by a pair of helices (49). Arora and colleagues have made some progress in this area by incorporating a chemical linker to bridge two helices that could stabilize a coiled-coil structure of the peptides (50, 51).

Previously, we observed that our lead peptide (SP1) was able to enter cells, using confocal microscopy to confirm the presence of FAM-labeled peptide inside the cells (34). Unfortunately, the modifications made to the peptide to improve affinity and solubility meant that the peptides were no longer able to enter cells (data not shown); therefore, their activity in cell culture could not be assessed. It has been shown previously that stapled peptides need to form an amphipathic helix in order to enter cells (52), and the amphipathicity of our peptide was disrupted by the inclusion of the solubilizing salt bridges. Amphipathicity *versus* solubility is likely to be a challenge when designing stapled peptides; however, a cell-penetrating peptide (CPP) sequence could be used to facilitate cell entry.

Like Ras proteins, the Ral GTPases are challenging drug targets, and there are currently no well-validated Ral inhibitors that can be used to study Ral activity or to be used as a treatment for Ras-mutant cancers. The Theodorescu group previously identified a small-molecule inhibitor that stabilized the inactive, GDP-bound form of Ral proteins through binding an allosteric site (29). This molecule was able to inhibit Ral activation and inhibited tumor growth in a mouse model of lung cancer. However, independent investigations comparing the effects of the inhibitor with Ral knockdown with siRNA showed that the inhibitor caused off-target effects in platelets (53). The covalent inhibitors that have been developed for K-Ras G12C offer great promise for the direct inhibition of this Ras mutant; however, most Ras mutants found in cancer, like the Ral proteins, lack an accessible cysteine that can be exploited for inhibition. However, Bum-Erdene *et al.* (30) have very recently demonstrated that Tyr82 of Ral, located within the effector-binding site, can be modified by aryl sulphonyl electrophiles. The compounds they developed are currently not suitable for animal trials due to their low affinity and poor serum stability but this work demonstrates a novel approach to target the Ral GTPases that could be incorporated to improve our peptide inhibitors. In the wild-type RLIP76 RBD/RalB complex, His413 of the RBD forms a hydrogen bond with Tyr82 of Ral (Fig. 11). In our peptides His413 is not included in the sequence; therefore, the hydroxyl group of Tyr82 is presumably not involved in the peptide interaction but remains proximal to the binding site. This therefore offers an enticing opportunity to convert stapled peptides into covalent inhibitors targeting Ral proteins, as a reactive warhead could be installed on the side of the peptide directed toward Tyr82. Covalent inhibitors require a lower binding affinity for sustained inhibition due to irreversible modification of the target. As our peptides display excellent selectivity for the Ral proteins, they could be highly effective as covalent inhibitors with limited off-target effects.

The peptides could also form the basis for proteolysis targeting chimeras (PROTACs), molecules designed to target a protein for degradation: this catalytic mechanism requires a lower target affinity than is needed for sustained inhibition. Degradation of Ral proteins offers an interesting opportunity for the inhibition of oncogenic Ras signaling, as several Ras-mutant cancers require Ral activity for survival, unlike nontransformed cells (24).

In conclusion, the second-generation peptides we have produced demonstrate far superior selectivity for Ral proteins and improved physical properties compared with our previous lead peptide. With the potential for further modifications to aid their activity, they represent a further advance toward inhibition of the Ral GTPases.

## Experimental procedures

### Protein preparation

Proteins were expressed from pGEX vectors (Cytiva) as GST fusion proteins or from pMAT10 (35) as His_6_-MBP fusion proteins. The constructs expressing RalA (pMAT10, residues 1–184, Q72L) (36), RalB (pMAT10, 1–185, Q72L) (36), RLIP76 RBD (pGEX-HisP, 393–446, C411S) (54), RhoA (pGEX-2T, 1–186, F25N/Q63L) (55), Rac1 (pGEX-2T, 1–185, Q61L) (56), Cdc42 (pGEX-2T, 1–184, Q61L) (56), and K-Ras (pGEX-6P, 1–169) (38) were prepared, expressed, and cleaved from their tags as described previously. ^15^N-labeled RalB (1–185, Q72L) was prepared from pET16b in M9 minimal media supplemented with ^15^NH_4_Cl as described previously (36), without cleaving the His-tag.

### Nucleotide exchange

Ral proteins were labeled with [^3^H]GTP for use in SPAs as follows. [^3^H]GTP (0.15 mCi, PerkinElmer Life Sciences) was dried by centrifugal evaporation. Ral (0.7 mg) and 0.3 M (NH_4_)_2_SO_4_ were added in 140 μl of buffer (10 mM Tris-HCl, 150 mM NaCl, 1 mM DTT, pH 7.5). The mixture was incubated at 37 °C for 3 h, and unbound nucleotide was removed with Sephadex G25 spin columns (GE Healthcare). Small GTPases were exchanged for GMPPNP as described previously (57).

### CIS display

The peptide sequences encoding the RLIP76 RBD (residues 423–446, C411S) were used in a CIS display affinity maturation selection. Construct preparation, biopanning reactions, next-generation sequencing, and output ranking were purchased from Isogenica (Chesterford Research Park). Biotinylated RalA·GMPPNP and RalB·GMPPNP were produced by incubating 1 mg of each protein with a 20X excess of biotin in amine-free buffer at room temperature for 30 min. Labeled protein was separated using a PD10 buffer exchange column following the manufacturer’s instructions. During the selection, certain residues were restricted to a subset of sidechains: Leu423 and Ala438 were allowed to change to Asp, Glu, Lys, Asn, Arg, Ser, Ala, Gly, and Thr, whereas Arg444 was restricted to His, Asn, Ser, Lys, Gln, and Arg. Substitution by any of the 20 amino acids was enabled at the other positions.

### Scintillation proximity assays

#### Direct-binding SPAs

RLIP76 RBD-His variants (80 nM) were immobilized on Protein A SPA fluoromicrospheres (Perkin Elmer) *via* an anti-His antibody (H1029, Sigma-Aldrich) in 50 mM Tris-HCl pH 7.5, 2 mM DTT, 1 mM MgCl_2_, and 0.2 mg/ml BSA. [^3^H]GTP·Ral proteins were titrated at the concentrations indicated in the results. Experiments were performed as described previously (36). The equilibrium binding constants (*K*_d_) for the effector–Ral interactions were determined by monitoring the SPA signal in the presence of varying concentrations of [^3^H]GTP·Ral and fitted using nonlinear regression with the computer program Grafit.

#### Competition SPAs

Reaction mixtures were set up as described for direct measurements with the addition of [^3^H]GTP·RalA (100 nM) or [^3^H]GTP·RalB (250 nM). The effect of competition was assessed by measuring the SPA signal in the presence of increasing concentrations of peptides or proteins at the concentrations indicated in the results. The data were fitted to an appropriate binding isotherm as described previously (58).

### Circular dichroism

CD spectra were recorded at 1 nm intervals between 260 and 185 nm using an Aviv Model 410 CD spectrometer with a 1 mm path length quartz cuvette at 298 K. Three scans were recorded for each peptide or protein, the data were averaged, and the buffer background was subtracted. RLIP76 RBDs were measured at 0.2 mg/ml in 10 mM phosphate buffer, pH 7.3, and 150 mM NaF, while peptides were measured at 0.2 mg/ml in water. The helical content of each peptide and RBD was determined using the CDSSTR method with reference Set 3 and DichroWeb (59, 60, 61).

### Crystallization

Complexes of RalB·GMPPNP and RLIP76 RBD (HLR and SMLR mutants) were generated by incubating RalB·GMPPNP in the presence of excess RLIP76 RBD prior to purification on an S75 gel filtration column (GE Healthcare) in 20 mM Tris-HCl, pH 7.5, 100 mM NaCl, and 1 mM MgCl_2_.

Cocrystals of RalB·GMPPNP and RLIP76 RBD (HLR and SMLR mutants) were generated by screening the copurified complexes at 10 mg/ml with the pHClear Suite I screen (Qiagen). Crystallization trials were set up using the Mosquito robotics system (SPT Labtech). Drops were set up with 0.2 μl protein solution and 0.2 μl screen solution using the sitting drop vapor-diffusion method. Crystals formed in the condition containing 0.1 M Bicine, pH 9.0, 30% w/v polyethylene glycol 6000 at 20 °C. The crystals were frozen in liquid nitrogen prior to data collection. X-ray diffraction data was collected at the Diamond Light Source on beamlines IO3 and IO4 and processed using the pipedream package (Global Phasing Ltd). The structures were determined by molecular replacement using Phaser (62) from the CCP4 package (63) and were iteratively built and refined using Coot (64) and PHENIX (65). Coordinates have been deposited to the protein data bank under the accession codes 6ZQT (HLR mutant) and 6ZRN (SMLR mutant).

### Isothermal titration calorimetry

ITC data were collected using a MicroCal iTC200 calorimeter at 298 K in 50 mM Tris-HCl, pH 7.5, 150 mM NaCl, 1 mM MgCl_2_. RalB (40–200 μM, 8–10× cell concentration) was titrated into RLIP76 RBD variants (5–20 μM) in 19 × 2 μl additions with 120 s between injections. Control experiments were performed by titrating RalB (200 μM) into buffer. Data were fitted using MicroCal Origin 7.0 software using a single-site binding model.

### Peptide synthesis

Peptides with an amidated C-terminus were synthesized by standard Fmoc/tBu solid-phase chemistry on an automated peptide synthesizer (PTI Prelude) using Rink Amide MBHA resin (0.30 mmol/g loading, 500 mg, 0.15 mmol scale). Fmoc-(*S*)-pentenylalanine required for staple formation was manually coupled as follows. Fmoc-(*S*)-pentenylalanine (228 mg, 0.6 mmol, 4 eq), hexafluorophosphate azabenzotriazole tetramethyl uranium (HATU, 228 mg, 0.6 mmol, 4 eq), and *N,N*-diisopropylethylamine (DIPEA, 210 μl, 1.2 mM, 8 eq) were added to the deprotected resin in *N,N*-dimethylformamide (DMF, 5 ml) and incubated with shaking at room temperature for 1 h. The olefin hydrocarbon-substituted peptides were chemically stapled by metathesis reaction with 2 × 5 ml Grubbs’ first-generation catalyst (6 mM) in 1,2-dichloroethane at room temperature for 2 h with N_2_ sparging (66). FAM labeling was carried out using 5-carboxyfluorescein (282 mg, 0.75 mmol, 5 eq), hydroxybenzotriazole (HOBt, 230 mg, 1.5 mmol, 10 eq), and *N,N'*-Diisopropylcarbodiimide (DIC, 237 μl, 1.5 mmol, 10 eq) in DMF (5 ml). Reaction vessels were covered in foil and incubated with shaking at room temperature for at least 24 h. Peptide resins were washed extensively with dichloromethane, DMF, and diethyl ether and dried before cleaving from the resin with 89% trifluoroacetic acid (TFA), 5% triisopropylsilane (TIPS), 1.5% ethanedithiol (EDT), 1.5% water, 1.5% thioanisole, and 1.5% phenol for 4 h at room temperature. Peptides were purified by reversed-phase preparative HPLC (Waters X-Bridge, 19 × 250 mm, C18 OBD) and analyzed by LC/MS (Agilent Polaris C8A, 2.1 × 50 mm), both systems eluting gradients of acetonitrile (0.1% v/v TFA) against water (0.1% v/v TFA). See Table S2 for characterization data for all peptides synthesized. The peptides termed SP1, nonstapled, and HLR-SP1 were purchased from Eurogentec.

### Fluorescence polarization

FP experiments were performed on a BMG Labtech Pherastar fluorimeter at 298 K with excitation at 485 nm and emission at 520 nm as described previously (34), using 20 nM FAM-labeled peptide. Plates were read after 30 min incubation at room temperature. Data were fitted to a single-site binding model using nonlinear regression analysis in GraphPad prism 8.4 to obtain *K*_d_ values and their standard errors.

### ^1^H,^15^N HSQC NMR spectroscopy

Experiments were recorded on a Bruker AV800 at 298 K using 100 μM ^15^N-labeled RalB·GMPPNP in 50 mM Tris-HCl pH 7.5, 100 mM NaCl, 1 mM MgCl_2_, 10% D_2_O. For the titration experiments, 0.25, 0.5, 1.0, 1.25, and 2.0 equivalents of HLR-sol were added to the protein solution, and spectra were recorded after each peptide addition. Chemical shift perturbations (δ) were calculated using the following equation: $\delta =\;\sqrt{\delta_{1H}^{2}+\left(0.15\delta_{15N}\right)}$, where δ^1^H and δ^15^N are the chemical shift changes for the ^1^H and ^15^N dimensions, respectively. NMR data were processed using the AZARA package (Wayne Boucher, University of Cambridge) and analyzed using CCPN ANALYSIS (67).

### Coimmunoprecipitation assays

3 × 10^6^ HEK293T cells were seeded in 10 cm dishes 48 h before the end point, in DMEM supplemented with 10% FBS. For RalB/RLIP76 coimmunoprecipitations cells were transfected with GFP (1 μg), flag-RLIP76 (5 μg), and V5-RalB Q72L (5 μg) or GFP only (11 μg) 24 h before the end point, while for RalB/Sec5 coimmunoprecipitations cells were transfected with GFP (1 μg) and V5-RalB Q72L (5 μg) or GFP only (6 μg) 24 h before the endpoint. Dishes were lysed in 1 ml lysis buffer (50 mM Tris-HCl pH 7.8, 150 mM NaCl, 1% Triton, 1 mM EDTA, 1 mM sodium orthovanadate, 1 mM β-glycerophosphate, and protease inhibitor cocktail (Sigma-Aldrich). Lysates (1 ml) were centrifuged at 17,000*g* for 20 min and added to Protein G Dynabeads (30 μl, Invitrogen) preincubated with anti-V5 antibody (1 μg, R960-25, Invitrogen). Peptides, at the concentrations indicated in the results, were added and incubated with rotation for 1 h at 4 °C. Precipitated complexes were washed with lysis buffer (3 × 500 μl). Samples were resolved by SDS-PAGE and transferred to PVDF membranes (Immobilon-P). Membranes were probed by immunoblotting with the following primary antibodies: anti-V5-HRP (R961-25, Invitrogen), anti-flag-HRP (A8592, Sigma-Aldrich), and anti-Sec5 (EPR9420, Abcam).

## Data availability

Accession codes: Coordinates have been deposited to the protein data bank under the accession codes 6ZQT (HLR mutant) and 6ZRN (SMLR mutant). All relevant data associated with the article are available upon request from the corresponding author.

## Conflict of interest

The authors declare that they have no conflicts of interest with the contents of this article.
