# Supplementary material for: Affinity maturation of the RLIP76 Ral binding domain to inform the design of stapled peptides targeting the Ral GTPases
Source: J Biol Chem. 2020 Nov 23;296:100101. doi: 10.1074/jbc.RA120.015735 (PMC7949049; doi:10.1074/jbc.RA120.015735)
Supplement: Supplementary Figures and Tables [file mmc1.pdf]

Supporting Information

Maturation of the Ral binding domain of RLIP76 to inform the design of stapled peptides targeting the Ral GTPases

Catherine A. Hurd[1], Paul Brear[1], Jefferson Revell[2], Sarah Ross[3], Helen R. Mott[1*], Darerca Owen[1*]

Table S1: Affinity measurements for second-generation peptides binding to a panel of small GTPases.

Table S2: Characterization data for all synthesized peptides.

Figure S1. Binding of RalA to the wild-type and LTTLR-mutant RLIP76 RBD measured by direct SPA.

Figure S2. Binding of RalA proteins to wild-type and WDASQSR-mutant RLIP76 RBDs measured by direct SPA.

Figure S3. CD spectra of the RLIP76 RBD variants.

Table S1: Affinity measurements for second-generation peptides binding to a panel of small GTPases.

**GTPase**	**K_d (μM)[a]**	
	**SP1**	**HLR-SP1**
RalA	17.2 ± 9.2	4.68 ± 0.64
RalB	4.70 ± 2.04	0.159 ± 0.047
K-Ras	8.18 ± 1.99	1.06 ± 0.20
Cdc42	13.0 ± 7.4	6.11 ± 1.67
RhoA	0.0338 ± 0.0054	0.0125 ± 0.0022

[a] Standard error from curve fitting.

Table S2: Characterization data for all synthesized peptides.

Peptide name	Sequence[a]	Expected masses[b]	Masses found[b]	R_t (min)
HLR-sol	FAM-PEG-LXKEHXLWEELRIKTAERRKKREA	MW – 3587.2 1794.6/1196.7/897.8/718.4	1196.7/897.8/718.4	4.6
L-sol	FAM-PEG-LXKEEXLWEELRIKTAEKRKKREA	MW – 3551.1 1776.6/1184.7/888.8/711.2	1184.7/888.7/711.2	5.0
wt-sol	LXKEEXLWEEQRIKTAEKRKKREA	MW – 3048.6 1525.3/1017.2/763.2/610.7	1525.2/1016.9/763.1/610.8	3.9
W430A-sol	LXKEEXLAEEQRIKTAEKRKKREA	MW – 2933.7 1467.7/978.8/734.4/587.7	1467.6/978.7/734.3/587.8	3.7

[a] X = (*S*)-pentenylalanine, FAM = 5-carboxyfluorescein, PEG = polyethylene glycol linker, amino-4,7-dioxanonanoic acid.
[b] Expected and observed masses from LC-MS analysis.

Supporting Information Figure Legends

Figure S1. Binding of RalA to the wild-type and LTTLR-mutant RLIP76 RBD measured by direct SPA. The indicated concentrations of [^{3}H]GTP-labelled RalA were incubated with His-tagged wild-type or LTTLR-mutant RLIP76 RBD (80 nM). The signal was corrected by subtraction of the background signal from parallel measurements containing no RLIP76 RBD. The data and curve fits are displayed as a proportion of this maximal signal. The data were fitted to a direct binding isotherm to give an apparent K_d value and the maximum signal at saturating Ral concentrations: wild-type, 306 ± 59 nM; LTTLR mutant, no fit could be obtained as binding was too weak. n = 2.

Figure S2. Binding of RalA proteins to wild-type and WDASQSR-mutant RLIP76 RBDs measured by direct SPA. The indicated concentration of [^{3}H]GTP-labeled RalA was incubated with His-tagged wild-type or WDASQSR-mutant RLIP76 RBD (80 nM). The signal was corrected by subtraction of the background signal from parallel measurements containing no RLIP76 RBD. The data were fitted to a binding isotherm to give an apparent K_d value and the maximum signal at saturating Ral concentrations. The data and curve fits are displayed as a proportion of this maximal signal: K_d wild-type = 45.1 ± 6.4 nM; WDASQSR, no fit could be obtained as binding was too weak. n = 2.

Figure S3. CD spectra of the RLIP76 RBD variants. CD data are reported as mean residue ellipticity (deg cm^2 $dmol^{-1}$, θ) over the wavelength range 185-260 nm. The calculated helicity and ratio of the mean

residue ellipticity at 222 and 208 nm ($[\theta]_{222}/[\theta]_{208}$) are shown in the inset. Coiled-coils have a $[\theta]_{222}/[\theta]_{208}$ value close to 1.0, while isolated α-helices have values closer to 0.8.

Figure S4. Binding of SP1 and HLR-SP1 peptides to a panel of small GTPases. The peptide sequences are displayed. FP data for direct binding of 20 nM FAM-labelled SP1 (**A**) and HLR-SP1 (**B**) to varying concentrations of indicated small GTPases. Data were fitted to a single-site binding model using non-linear regression analysis in GraphPad Prism, and the calculated K_d values are listed in Table S1. Data and curve fits are displayed as a percentage of the calculated saturated FP signal in each assay. $n \geq 2$ for all conditions, and individual results are displayed as symbols. The residues involved in the hydrocarbon staple are denoted by a red X, the residues that differ between SP1 and HLR-SP1 are shaded green in panel B.

Figure S1

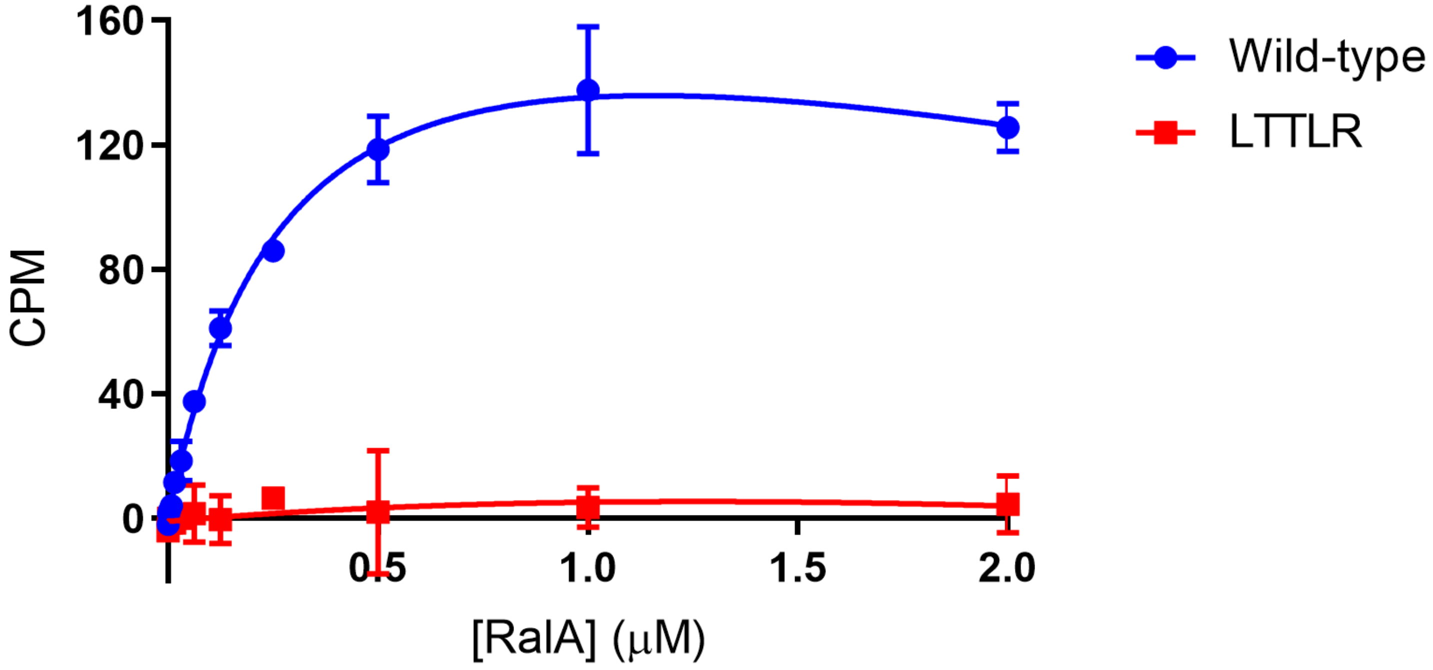

Figure S2

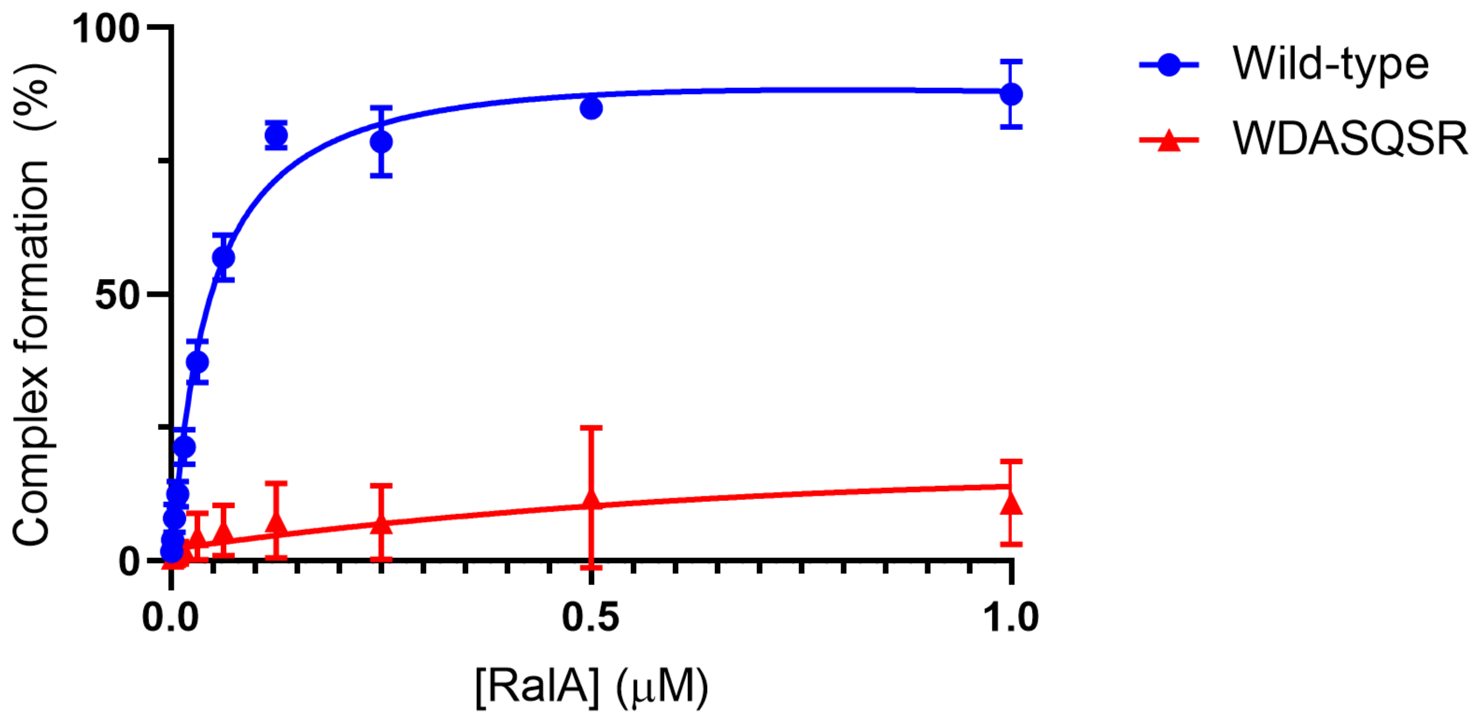

Figure S3

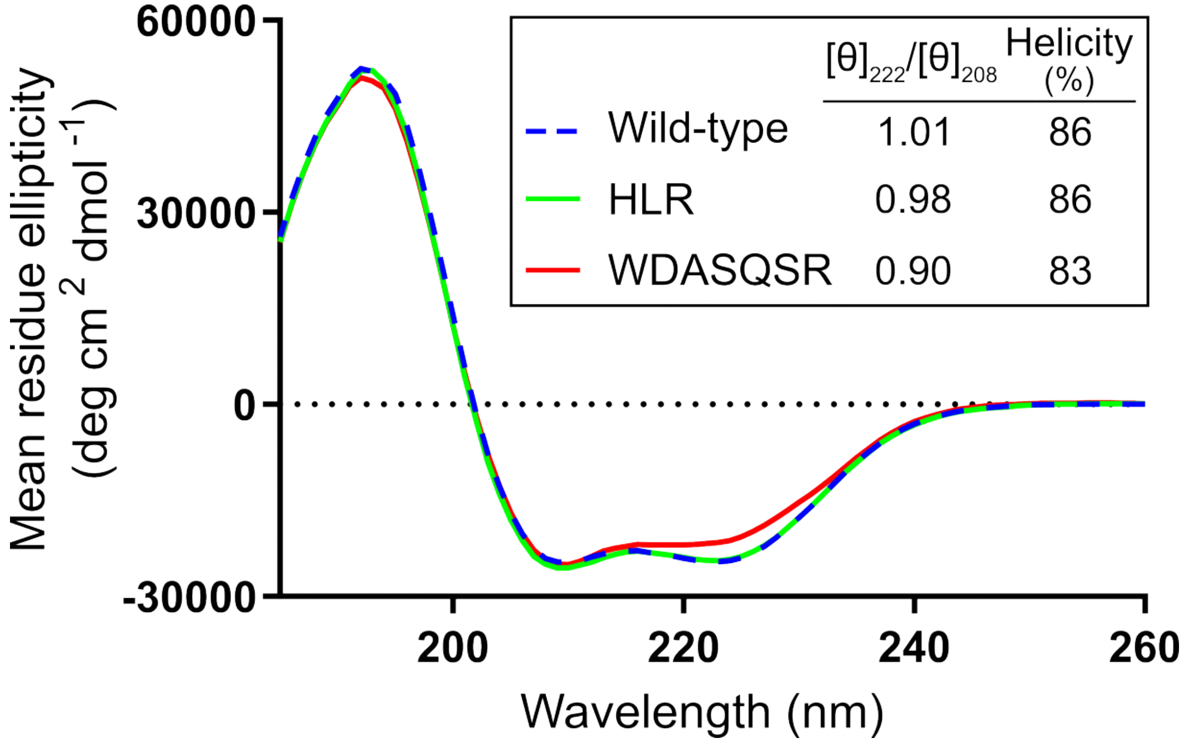

60000
30000
0
-30000
Mean residue ellipticity
(deg cm 2 dmol -1)
200
220
240
260
Wavelength (nm)
[θ]222/[θ]208
Helicity (%)
Wild-type 1.01 86
HLR 0.98 86
WDASQSR 0.90 83

Figure S4

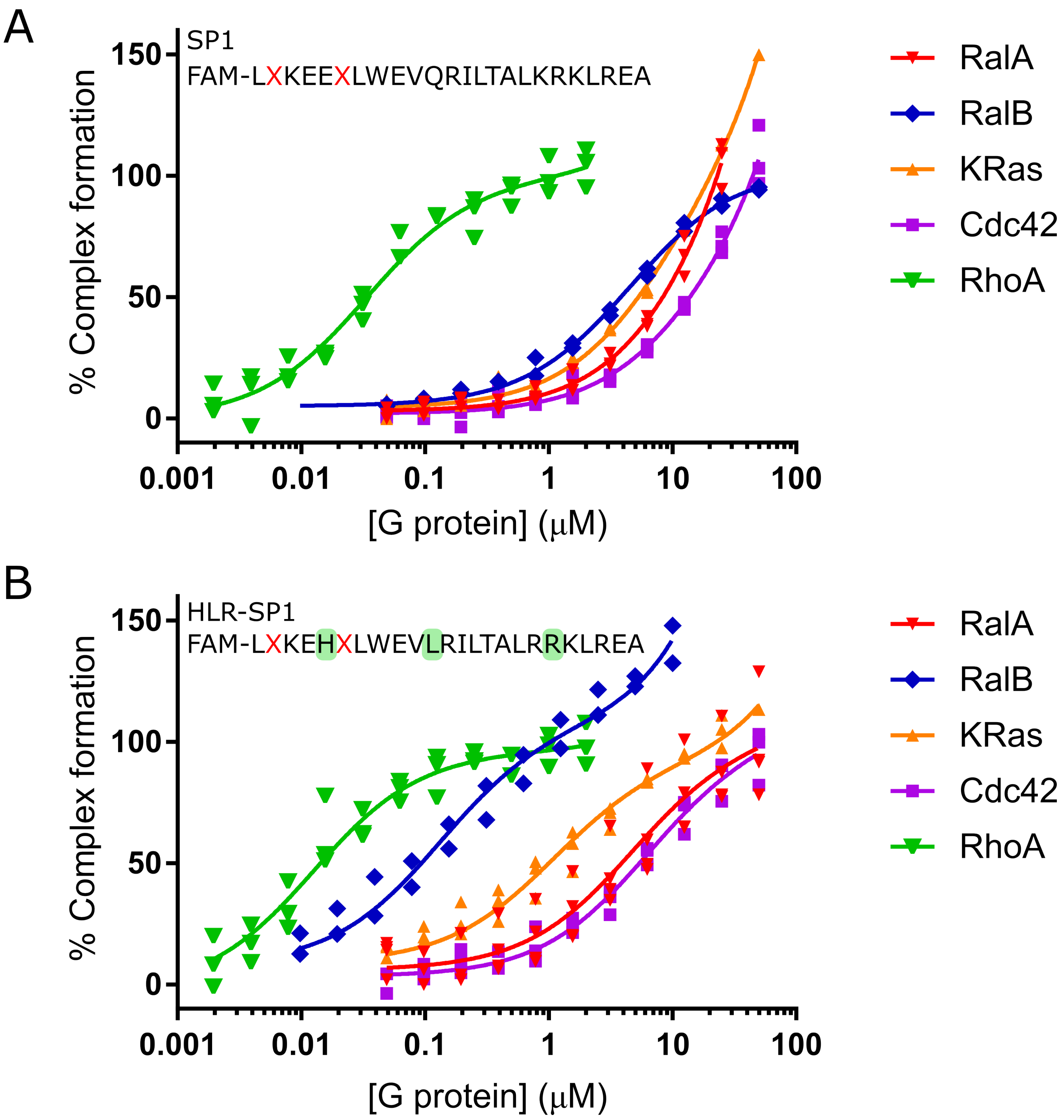
A
SP1
FAM-LXKEEXLWEVQRILTALKRKLREA
% Complex formation
150
100
50
0
0.001
0.01
0.1
1
10
100
[G protein] (μM)
RalA
RalB
KRas
Cdc42
RhoA
B
HLR-SP1
FAM-LXKEHXLWEVLRILTALRRKLREA
% Complex formation
150
100
50
0
0.001
0.01
0.1
1
10
100
[G protein] (μM)
RalA
RalB
KRas
Cdc42
RhoA
